# Nanoporous Gold Catalyst for the Oxidative N‐Dealkylation of Drug Molecules: A Method for Synthesis of N‐Dealkylated Metabolites

**DOI:** 10.1002/cmdc.202200040

**Published:** 2022-04-05

**Authors:** Ali Alipour Najmi, Elchin Jafariyeh‐Yazdi, Mojgan Hadian, Jos Hermans, Rainer Bischoff, Jun Yue, Alexander Dömling, Arne Wittstock, Hjalmar P. Permentier

**Affiliations:** ^1^ Department of Analytical Biochemistry Groningen Research Institute of Pharmacy University of Groningen A Deusinglaan 1 9713 AV Groningen The Netherlands; ^2^ Department of Chemical Engineering Engineering and Technology Institute Groningen University of Groningen 9747 AG Groningen The Netherlands; ^3^ Department of Drug Design University of Groningen A Deusinglaan 1 9713 AV Groningen The Netherlands; ^4^ Institute of Applied and Physical Chemistry and Center for Environmental Research and Sustainable Technology University Bremen Leobener Str. 6 28359 Bremen Germany

**Keywords:** nanoporous gold, N-dealkylation, drug metabolite, metabolite synthesis, catalysis

## Abstract

A novel method for the selective catalytic N‐dealkylation of drug molecules on a nanoporous gold (NPG) catalyst producing valuable N‐dealkylated metabolites and intermediates is described. Drug metabolites are important chemical entities at every stage of drug discovery and development, from exploratory discovery to clinical development, providing the safety profiles and the ADME (adsorption, distribution, metabolism, and elimination) of new drug candidates. Synthesis was carried out in aqueous solution at 80 °C using air (oxygen source) as oxidant, in single step with good isolated yields. Different examples examined in this study showed that aerobic catalytic N‐dealkylation of drug molecules on NPG has a broad scope supporting N‐deethylation, N‐deisopropylation and N‐demethylation, converting either 3° amines to 2° amines, or 2° amines to 1° amines.

## Graphical abstract

**Facilitating drug discovery**: We describe a novel method for the selective catalytic N‐dealkylation of drug molecules on a nanoporous gold (NPG) catalyst producing valuable N‐dealkylated metabolites and intermediates. Various examples examined in this study showed that aerobic catalytic N‐dealkylation of drug molecules on NPG has a broad scope, supporting N‐deethylation, N‐deisopropylation and N‐demethylation, converting either 3° amines to 2° amines, or 2° amines to 1° amines.

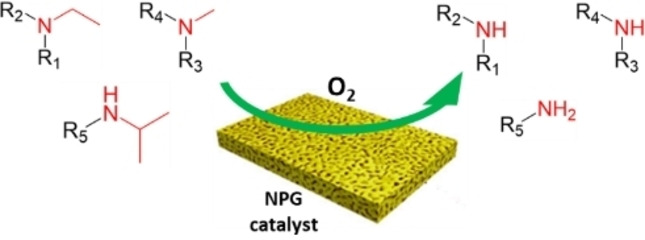

## Introduction

Drug molecules undergo various chemical modifications in the human body following administration, resulting in metabolites which can have toxicological and functional characteristics that are distinct from their parent drugs.^[1]^ The chemical modifications of drug metabolism are mainly carried out by members of the Cytochrome P450s (CYP) enzyme family in the human liver.^[2]^ Drug metabolites are valuable chemicals and play an integral part in the development of safe and effective pharmaceuticals, since they are critical for ADME (adsorption, distribution, metabolism, and excretion) studies during the early stages of drug discovery and development.[^3^, ^4^] The synthesis of metabolites on a preparative scale is important to perform toxicology, pharmacokinetics, pharmacodynamics, and bioavailability studies.^[5]^ Moreover, the elucidation of chemical structures and safety testing of metabolites are mandated by governmental regulators such as the U.S. Food and Drug Administration (FDA).[^6^, ^7^, ^8^] Drug metabolism studies are generally performed *in vivo* (e. g. animal models) or *in vitro* (e. g. perfused organs, liver microsomes) but these methods are not suitable to produce sufficient quantities of metabolites for their follow‐up studies. The isolation and purification of metabolites from these complex biological matrices is a challenging multi‐step and high‐cost process.[^2^, ^4^, ^9^] Although liquid chromatography‐mass spectrometry (LC‐MS) is the main technique for the elucidation of metabolite chemical structures in early stage studies, MS has inherent ambiguity in assigning regio‐ and stereo‐chemistry of modifications.[^4^, ^6^] Therefore, the development of synthetic routes based on plausible chemical modifications obtained from LC‐MS is a time‐ and resource‐intensive process especially when the synthesized chemical entities fail to match the proposed metabolite structure, making the chemical synthesis of drug metabolites a notoriously challenging task.[^6^, ^10^] A range of electrochemical,[^11^, ^12^] catalytic[^6^, ^13^, ^14^, ^15^] or enzymatic[^5^, ^10^, ^16^] methods have been reported as possible solutions for the efficient synthesis of metabolites.

Nanoporous gold (NPG) is a bicontinuous structure formed by the dealloying of an AuM alloy in which M is a less noble metal such as Ag or Cu. NPG has attracted considerable attention in the 21^st^ century for various applications such as sensors and heterogeneous catalysis.[^17^, ^18^, ^19^] Catalytic conversion of CO to CO_2_ and the highly selective oxidation of methanol to methyl formate over NPG are important gas‐phase reactions.[^20^, ^21^, ^22^] Different studies have reported the successful application of NPG as a heterogeneous catalyst in the liquid‐phase, importantly for the oxidation of alcohols[^23^, ^24^] and the hydrogenation of alkynes,[^25^, ^26^, ^27^] among others.[^28^, ^29^, ^30^, ^31^, ^32^] Herein, we present a new application of nanoporous gold as a heterogeneous catalyst for the synthesis of N‐dealkylated metabolites and intermediates of various drug molecules. This synthesis method was carried out in aqueous conditions at 80 °C using air as oxidant.

## Results and Discussion

**Figure 1:**
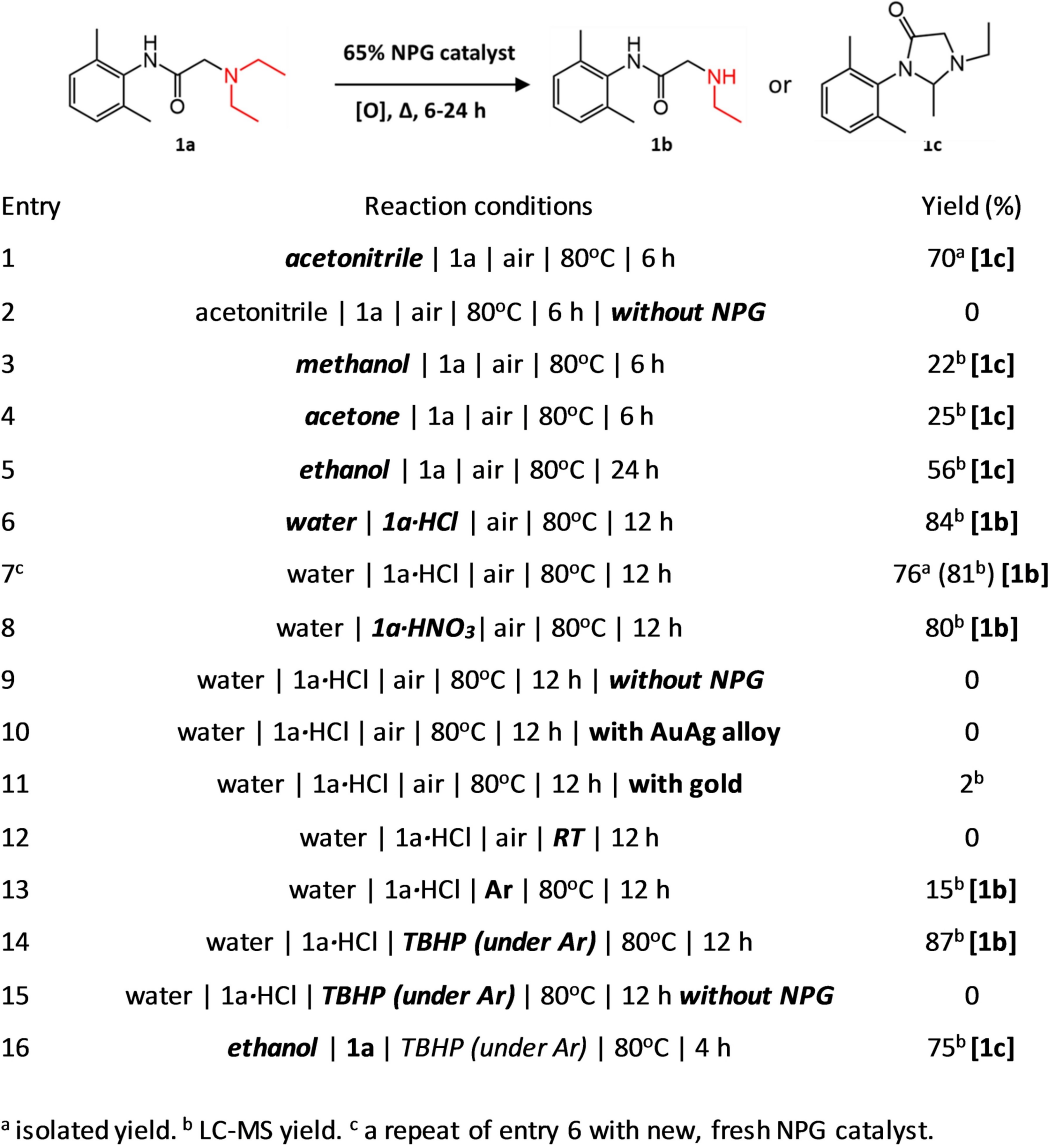
Catalytic oxidative N‐dealkylation of **1 a** to **1 b/1 c** using nanoporous gold (NPG) catalyst under different reaction conditions.

Following our years‐long interest in drug metabolism and in particular the N‐dealkylation of lidocaine **1 a**,[^12^, ^33^, ^34^, ^35^] we selected **1 a** as model drug. Initially, we performed the N‐dealkylation reaction of **1 a** in various organic solvents (MeCN, MeOH, EtOH and acetone) in the presence of air as oxygen source at 80 °C using 0.65 equivalent of nanoporous gold (NPG, 25 mg) catalyst (Figure 1, entries 1–5). To our surprise, the main product in the aerobic oxidation of **1 a** in the presence of NPG in these organic solvents was **1 c**, a cyclized form of **1 a** which has also been reported as a metabolite of lidocaine in humans.^[36]^ This reaction in acetonitrile led to 70 % isolated yield of **1 c** while in the absence of NPG no reaction was observed.

Changing the reaction medium from organic solvents to water led to the formation of the desired product, N‐dealkylated lidocaine **1 b**, as the main product. However, due to the limited solubility of **1 a** in water in its free‐amine form, a salt‐form of **1 a** was used in aqueous conditions (Figure 1, entries 6–8). The aerobic N‐dealkylation reaction of **1 a** in water led to 76 % isolated yield of **1 b**. LC‐MS analysis of the final reaction mixture showed **1 b** to be the major compound present in the reaction mixture and therefore **1 b** was isolated by liquid‐liquid extraction. No reaction was observed without NPG as catalyst in aqueous conditions either, confirming the key catalytic role of NPG in the transformation. Using an Au_30_Ag_70_ alloy (mother alloy of NPG) instead of NPG also did not lead to any conversion. Substitution of NPG with a piece of gold (25 mg) led to only 2 % conversion to **1 b** supporting the importance of the nanoporous structure for catalytic activity of NPG. There was no reaction at room temperature. Only 15 % yield was observed in the absence of air under argon atmosphere supporting the role of oxygen as oxidant. The limited amount of conversion under argon atmosphere can be contributed to the adsorbed oxygen onto the nanoporous gold surface.[^37^, ^38^] Importantly, adding tert‐butyl hydroperoxide (TBHP, 70 wt% in water, 1 equivalent) under argon atmosphere drove the reaction toward the formation of **1 b**, confirming the requirement of an oxidant in the catalytic conversion of **1 a** to **1 b**. Reaction with TBHP but in the absence of NPG did not lead to any N‐dealkylated product, while performing this reaction in organic solvents again led to the formation of **1 c** as the main product (Figure 1, entries 14–16).

**Figure 2:**
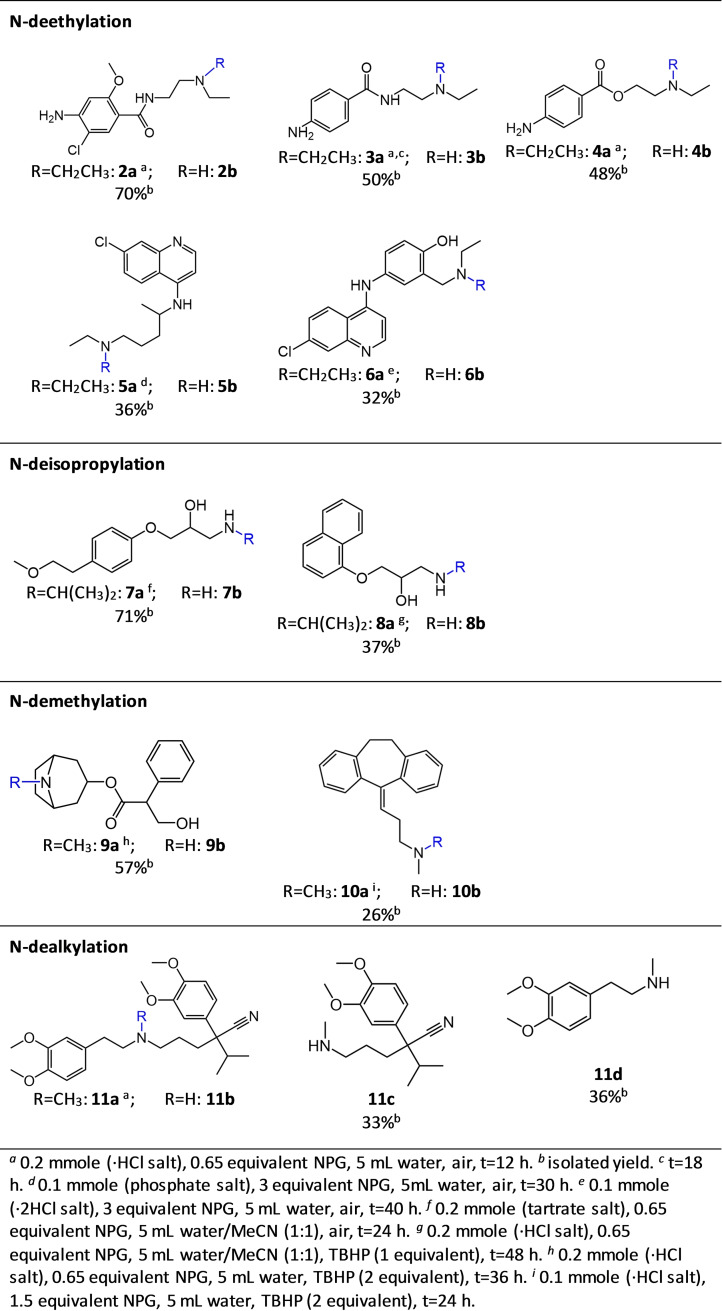
Scope of the catalytic oxidative N‐dealkylation of drug molecules using nanoporous gold catalyst.

Having studied different reaction conditions, we next applied the optimized conditions to other 2° or 3° amine‐containing drug molecules in order to examine the scope of the reaction (Figure 2). Oxidative catalytic N‐deethylation of compounds **2 a**–**6 a** converting a 3° amine to a 2° amine was successfully carried out using air as oxidant with isolated yields of up to 70 %. Compounds **7 a**–**8 a** were both converted to primary amines from their 2° amine structures following an N‐deisopropylation reaction.

Beside the importance of the N‐dealkylation reaction in the synthesis of drug metabolites, this reaction provides important intermediates for the semi‐synthesis of different medicines.[^39^, ^40^] For example, N‐dealkylation of atropine to noratropine is an important step for the semi‐synthesis of the bronchodilator ipratropium bromide which is registered in WHO's list of essential medicines.[^41^, ^42^] Oxidative catalytic N‐demethylation of atropine **9 a** in the presence of air as oxidant only led to 15 % conversion to **9 b**, while using TBHP as oxidant led to 57 % isolated yield. Furthermore, in the case of catalytic N‐demethylation of an aliphatic 3° amine to a 2° amine, conversion of compound **10 a** was negligible when using air as oxidant, similar to atropine, while using TBHP led to a low yield of **10 b** of 26 %. Interestingly, oxidative N‐dealkylation of verapamil **11 a** did not lead to norverapamil **11 b**, an N‐demethylated metabolite. This reaction produced two other N‐dealkylated structures of verapamil, **11 c** and **11 d** which are both reported as human metabolites.^[43]^

**Figure 3:**
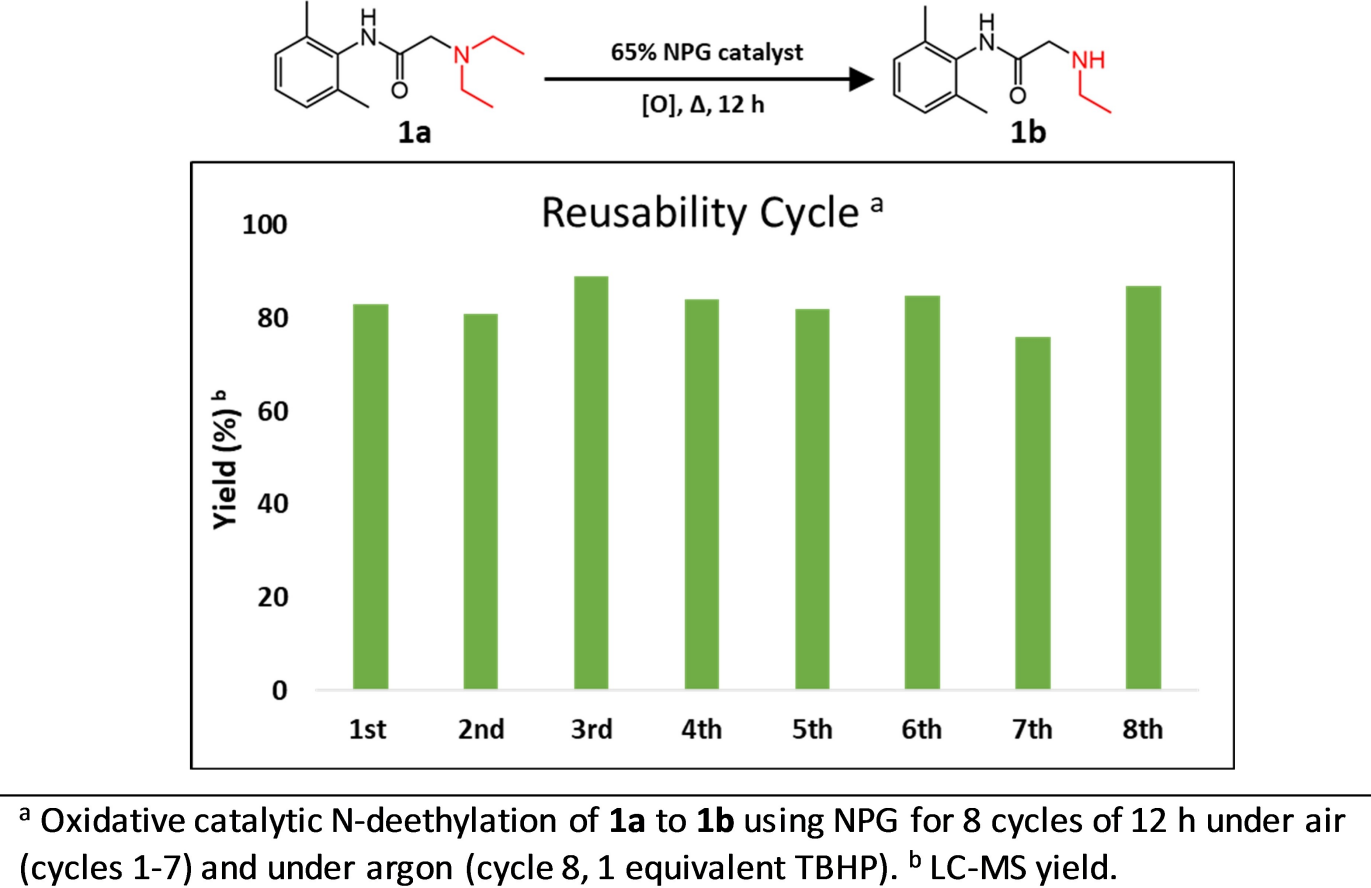
Reusability of NPG catalyst in 8 different cycles.

Finally, recovery and reusability of the NPG catalyst were investigated by its application for several cycles of oxidative N‐deethylation of lidocaine **1 a** (Figure 3). The change in catalytic activity of NPG during 8 cycles was found to be negligible.

## Conclusion

In summary, we introduce a new application of nanoporous gold (NPG) catalyst for oxidative N‐dealkylation of drug molecules in order to synthesize valuable drug metabolites and key intermediates for semi‐synthesis of different medicines. The reaction proceeds in aqueous conditions at elevated temperature using air (oxygen source) or TBHP as oxidant. The practicality of this method was shown by its broad scope, converting either a tertiary amine to a secondary amine or a secondary amine to a primary amine enabling N‐deethylation, N‐deisopropylation, and N‐demethylation. The recovery and reusability study showed that a single NPG catalyst can be used for a long period and multiple cycles. This work shows the potential of NPG catalyst for the synthesis of N‐dealkylated metabolites and intermediates providing the groundwork for further mechanistic studies to understand the catalytic pathways of the reaction and improve the reaction efficiency and catalyst‐to‐reactant ratio.

## Experimental Section

All solvents, chemicals and drug molecules were purchased from appropriate commercial suppliers. A Bruker Avance 500 spectrometer was used to record ^1^H‐ and ^13^C‐NMR spectra. A C_8_ reversed‐phase chromatography column (XBridge, Waters) was used in liquid chromatography‐mass spectrometry (LC‐MS) analysis with a Thermo Scientific TSQ Quantum Ultra mass spectrometer operating in positive mode using an electrospray ionization (ESI) ion source. High resolution MS spectra were recorded using either a Thermo Scientific Orbitrap Velos Pro or a Bruker maXis plus Q‐TOF mass spectrometer with ESI in positive mode. A preparative C_18_ column (XBridge BEH C_18_, OBD Prep Column, 130 Å, 5 μm, 10 mm×250 mm, Waters) or silica‐gel was used for chromatographic purification.

Nanoporous gold (NPG) samples were prepared by immersing a 4.8‐mm disc of gold‐silver alloy (30 at% Au and 70 at% Ag) with a thickness of 300 μm[^22^, ^44^] in concentrated nitric acid solution (100 mL, 96 h). After the dealloying process was finished, the dark black NPG sample was washed with excess water and acetone and then dried under reduced pressure overnight. All Au_30_Ag_70_ alloys were annealed at 600 °C for 18 h under nitrogen atmosphere using a Nabertherm tube furnace before dealloying.

General procedure for oxidative N‐dealkylation of drug molecules; 0.2 mmol of starting drug compounds (in HCl salt form) were dissolved in 5 mL water and then transferred to a home‐made 5‐mL reactor connected to a 4 bar air supply. The NPG sample was carefully placed inside a silver or gold gauze holder and then placed inside the reactor above a stirring magnet. If TBHP was used as oxidant, the gas supply was changed to argon or nitrogen. After 6–48 h, the gold or silver gauze including NPG was taken out and washed with 5 mL of acetone and then the wash solution was added to the reaction mixture. The final reaction mixture was dried under reduced pressure and purified over reversed‐phase column chromatography. The eluate (water/acetonitrile) was either dried under reduced pressure or directly lyophilized to obtain the N‐dealkylated product. In the case of 1b–2b, a two‐step liquid‐liquid extraction was used for product isolation; the dried crude was dissolved in 1 M HCl and washed with dichloromethane (DCM). The aqueous layer was then basified with NH_4_OH to pH 12–13 and extracted with DCM. Drying of the organic layer at reduced pressure yielded the isolated product.

## Conflict of interest

The authors declare no conflict of interest.

1

## Supporting information

As a service to our authors and readers, this journal provides supporting information supplied by the authors. Such materials are peer reviewed and may be re‐organized for online delivery, but are not copy‐edited or typeset. Technical support issues arising from supporting information (other than missing files) should be addressed to the authors.

Supporting Information

## Data Availability

The data that support the findings of this study are available from the corresponding author upon reasonable request.
