# Supplementary material for: Nanoporous Gold Catalyst for the Oxidative N‐Dealkylation of Drug Molecules: A Method for Synthesis of N‐Dealkylated Metabolites
Source: ChemMedChem. 2022 Apr 5;17(11):e202200040. doi: 10.1002/cmdc.202200040 (PMC9320976; doi:10.1002/cmdc.202200040)
Supplement: Supplementary file 1 — Supporting Information [file CMDC-17-0-s001.pdf]

ChemMedChem

Supporting Information

Nanoporous Gold Catalyst for the Oxidative N-Dealkylation of Drug Molecules: A Method for Synthesis of N-Dealkylated Metabolites

Ali Alipour Najmi, Elchin Jafariyeh-Yazdi, Mojgan Hadian, Jos Hermans, Rainer Bischoff, Jun Yue, Alexander Dömling, Arne Wittstock, and Hjalmar P. Permentier*

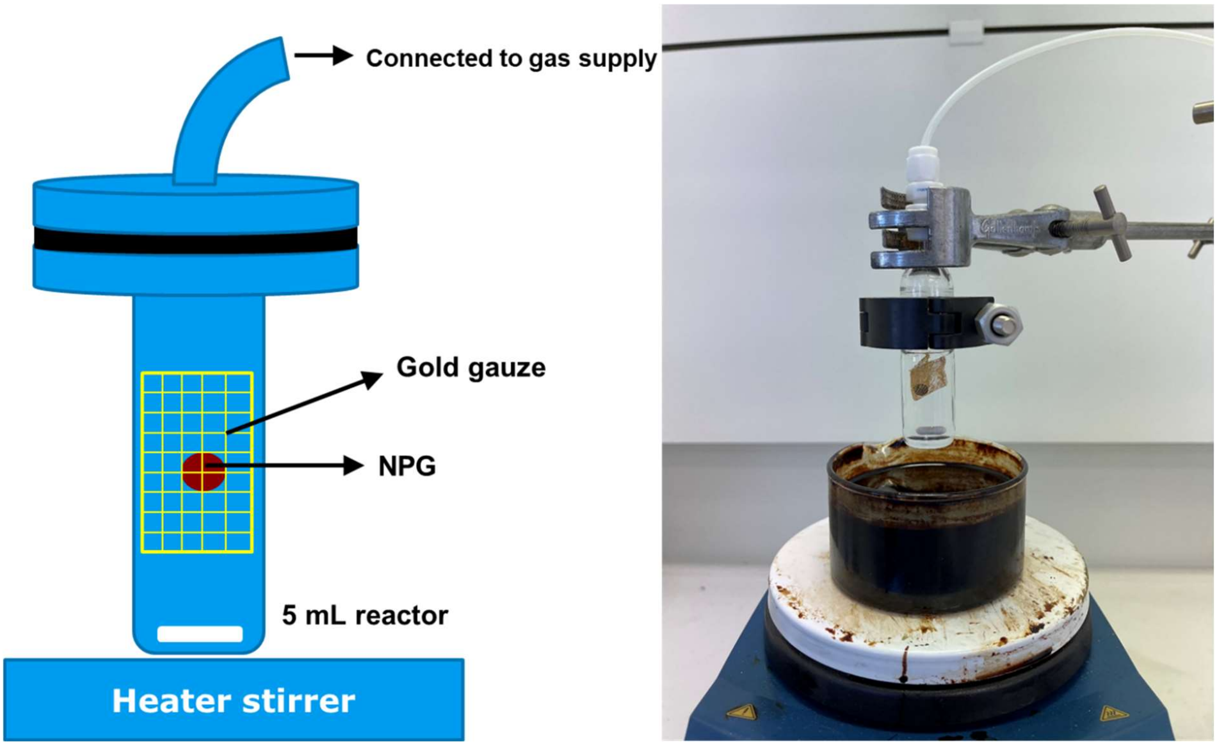

Figure SI_1, Left) a schematic of the designed 5-mL reactor including a gold gauze as NPG holder and a rubber-clamp system to make the system leak-free, right) a photograph of the reactor in practice.

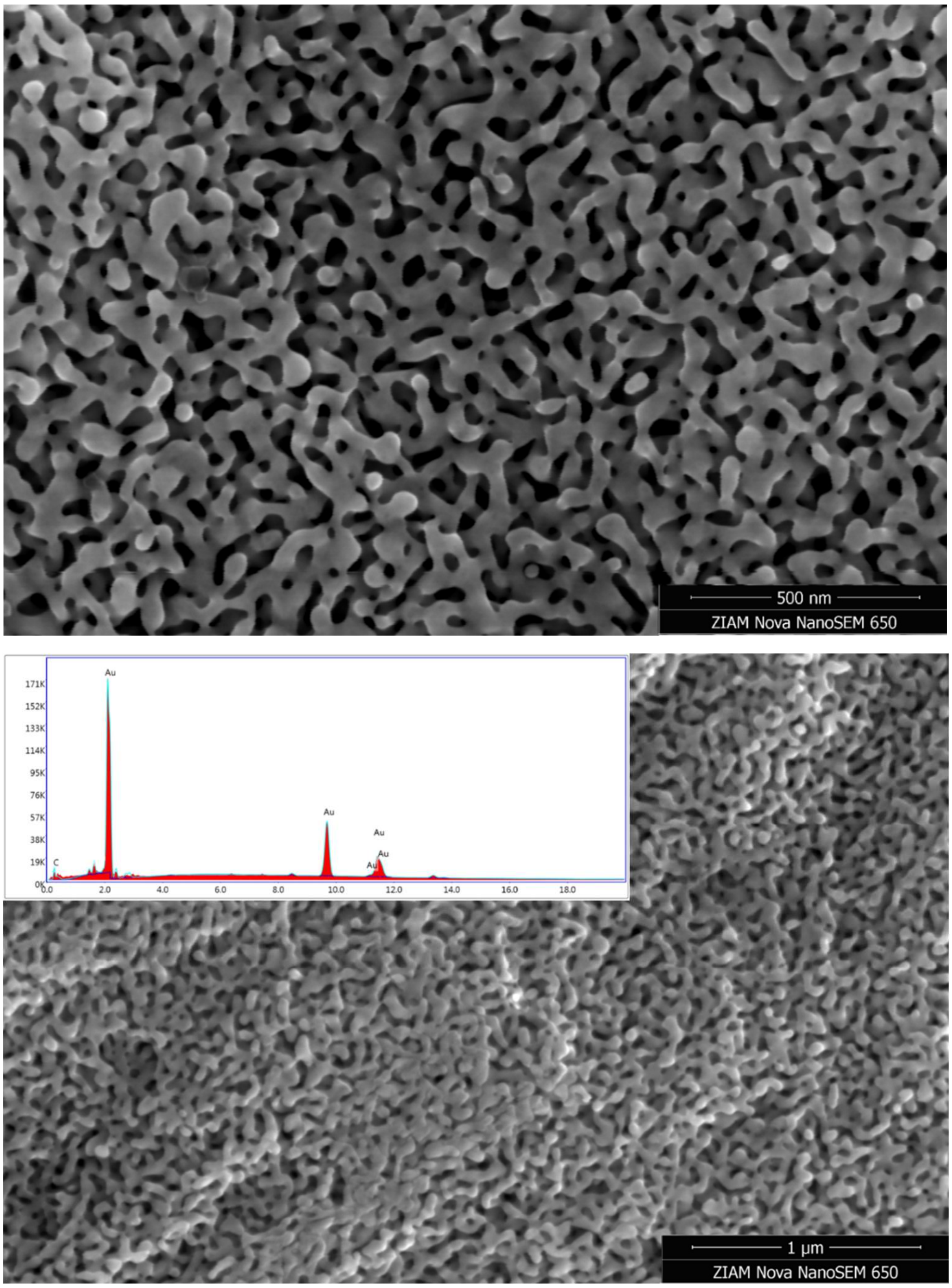

Figure SI_2, top) SEM image of a nanoporous gold sample, and bottom) SEM/EDX of the nanoporous gold sample with no more detectable Ag signal in the EDX measurement.

The specific surface area of nanoporous gold, S in [m^2/g], can be calculated from the ligament diameter d, the porosity factor for nanoporous gold C, and the bulk density of gold ρ using the equation $S = (C/\rho d)$ in which C=0.37 and ρ=19.8 g/cm^3 [1–3]. We used ImageJ software for image processing. The ligament size of NPG samples was 48.5 nm, determined by averaging 200 ligament size measurements. Therefore, using the equation above, the specific surface area is calculated to be 3.83 m^2/g.

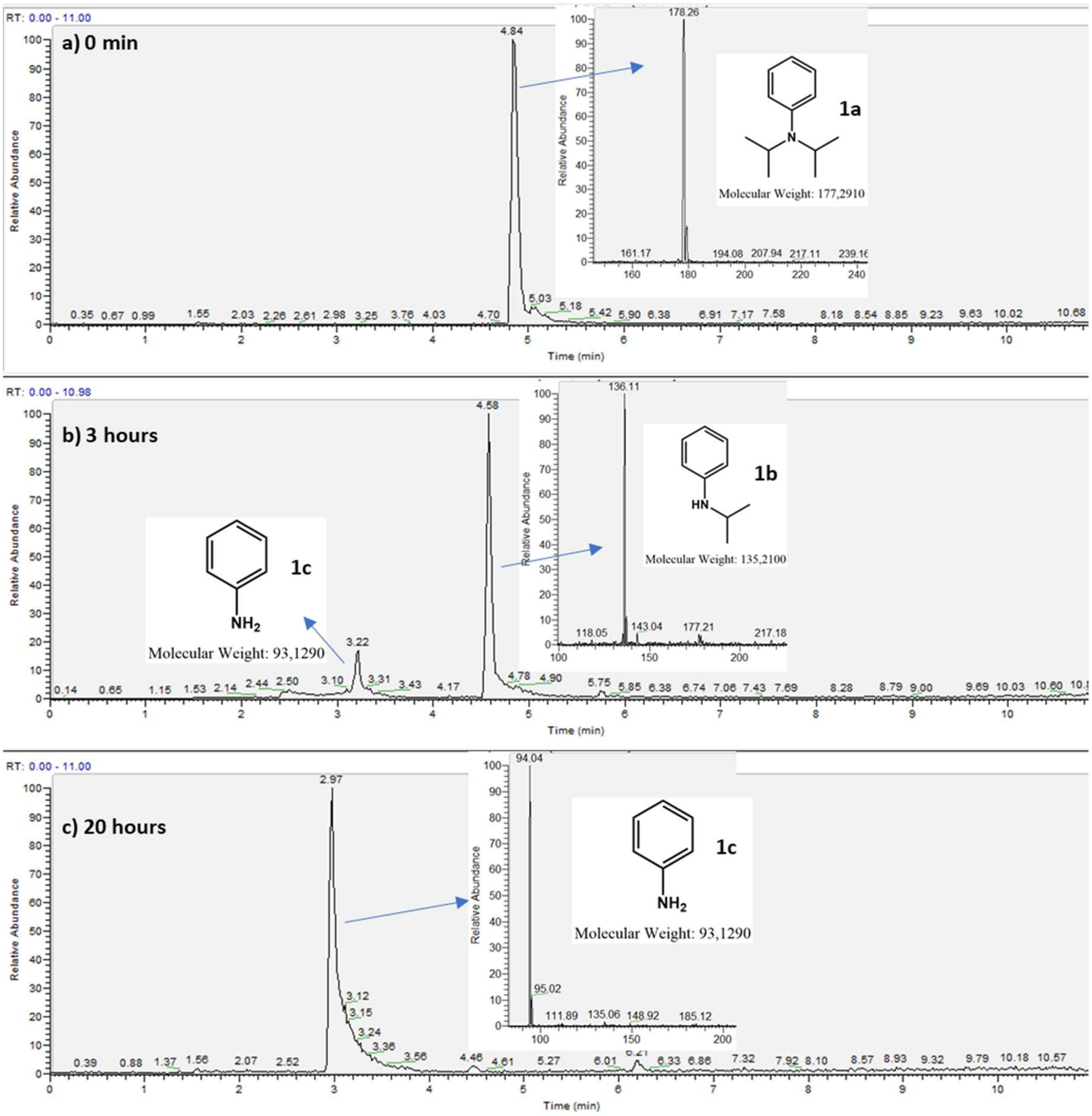

Figure SI_3, LC-MS chromatograms of reaction solutions of oxidative N-dealkylation of N,N-diisopropylaniline using NPG catalyst sampled at different time points. Reaction conditions: 0.1 mmole reactant, 5 mL solvent (1:1 water/MeCN), 80°C, under 4 bar air, 0.15 mmole NPG catalyst.

HRMS, ^{1}H and ^{13}C NMR of compounds

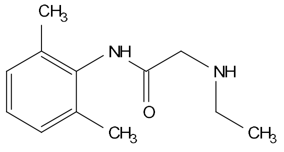

1b

^{1}H NMR (500 MHz, Chloroform-*d*) δ 8.86 (s, 1H), 7.13 – 7.08 (m, 3H), 3.48 – 3.45 (m, 2H), 2.80 (q, *J* = 7.1 Hz, 2H), 2.25 (s, 6H), 1.73 (s, 1H), 1.19 (t, *J* = 7.2 Hz, 3H). **^{13}C NMR (126 MHz, $CDCl_3$)** δ 170.27, 135.09, 133.88, 128.20, 127.06, 52.56, 44.83, 18.53, 15.49. **HRMS:** Calculated for $C_{12}H_{19}N_2O$ [M+H$^+$]=207.1492, observed = 207.1485.

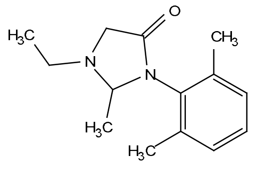

1c

^{1}H NMR (500 MHz, Chloroform-*d*) δ 7.27 – 7.05 (m, 3H), 4.48 (qt, *J* = 5.8, 1.6 Hz, 1H), 3.80 (dd, *J* = 14.7, 1.3 Hz, 1H), 3.20 (dd, *J* = 14.7, 1.9 Hz, 1H), 2.95 (dq, *J* = 11.6, 7.4 Hz, 1H), 2.49 (dq, *J* = 11.7, 7.0 Hz, 1H), 2.29 (s, 3H), 2.24 (s, 3H), 1.25 – 1.12 (m, 6H). **^{13}C NMR (126 MHz, $CDCl_3$)** δ 170.30, 138.06, 135.86, 133.16, 128.90, 128.76, 128.49, 77.28, 55.30, 47.61, 18.88, 18.76, 18.39, 13.49. **HRMS:** Calculated for $C_{14}H_{21}N_2O$ [M+H$^+$]=233.1648, observed = 233.1635.

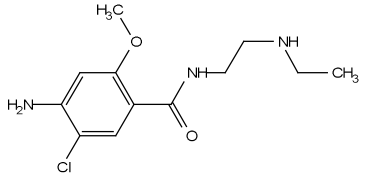

2b

^{1}H NMR (500 MHz, Chloroform-*d*) δ 8.09 – 8.03 (m, 2H), 6.29 (s, 1H), 4.58 (s, 2H), 3.86 (s, 3H), 3.52 (q, *J* = 5.9 Hz, 2H), 2.83 (t, *J* = 6.1 Hz, 2H), 2.69 (q, *J* = 7.2 Hz, 2H), 1.80 (s, 1H), 1.12 (t, *J* = 7.1 Hz, 3H). **^{13}C NMR (126 MHz, $CDCl_3$)** δ 164.81, 157.54, 146.85, 132.89, 112.34, 111.37, 97.79, 56.01, 48.60, 43.78, 39.63, 15.34. **HRMS:** Calculated for $C_{12}H_{19}ClN_3O_2$ [M+H$^+$]=272.1160, observed = 272.1148.

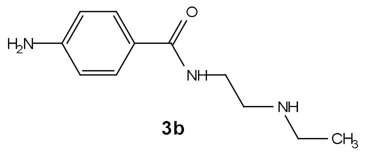

3b

^{1}H NMR (500 MHz, Methanol-*d*$_4$) δ 7.67 – 7.60 (m, 2H), 6.72 – 6.66 (m, 2H), 3.51 (t, *J* = 6.4 Hz, 2H), 2.84 (t, *J* = 6.3 Hz, 2H), 2.72 (q, *J* = 7.2 Hz, 2H), 1.16 (t, *J* = 7.2 Hz, 3H). **^{13}C NMR (126 MHz, Methanol-*d*$_4$)** δ 170.70, 153.22, 129.90, 123.01, 114.66, 49.64, 44.49, 40.03, 14.46. **HRMS:** Calculated for $C_{11}H_{18}N_3O$ [M+H$^+$]=208.1442 , observed =208.1444.

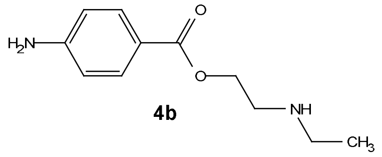

4b

^{1}H NMR (500 MHz, Methanol-*d*$_4$) δ 7.92 – 7.82 (m, 2H), 6.79 – 6.69 (m, 2H), 4.58 – 4.47 (m, 2H), 3.44 (t, *J* = 5.1 Hz, 2H), 3.18 (q, *J* = 7.3 Hz, 2H), 1.38 (t, *J* = 7.3 Hz, 3H). **^{13}C NMR (126 MHz, Methanol-*d*$_4$)** δ 168.01, 153.63, 132.88, 118.62, 115.15, 60.89, 47.62, 44.54, 11.45. **HRMS:** Calculated for $C_{11}H_{17}N_2O_2$ [M+H$^+$]=209.1285, observed =209.1284.

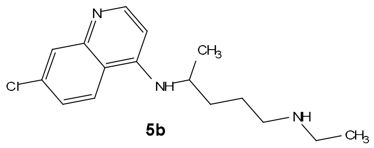

5b

^{1}H NMR (500 MHz, Chloroform-*d*) δ 8.48 (d, *J* = 5.4 Hz, 1H), 7.92 (d, *J* = 2.2 Hz, 1H), 7.76 (d, *J* = 8.9 Hz, 1H), 7.31 (dd, *J* = 8.9, 2.2 Hz, 1H), 6.38 (d, *J* = 5.5 Hz, 1H), 5.67 (d, *J* = 7.2 Hz, 1H), 3.72 – 3.65 (m, 1H), 2.69 – 2.63 (m, 4H), 2.44 (s, 2H), 1.82 – 1.74 (m, 1H), 1.72 – 1.59 (m, 3H), 1.30 (d, *J* = 6.3 Hz, 3H), 1.14 (t, *J* = 7.1 Hz, 3H). **^{13}C NMR (126 MHz, Chloroform-*d*)** δ 151.92, 149.28, 149.24, 134.74, 128.56, 124.91, 121.52, 117.40, 99.11, 49.15, 48.34, 44.12, 34.02, 26.30, 20.13, 14.98. **HRMS:** Calculated for $C_{16}H_{23}ClN_3$ [M+H$^+$]=292.1570, observed =292.1570.

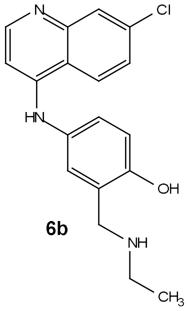

6b

^{1}H NMR (500 MHz, Methanol-*d*$_4$) δ 8.30 (d, *J* = 5.6 Hz, 1H), 8.26 (d, *J* = 9.0 Hz, 1H), 7.83 (d, *J* = 2.2 Hz, 1H), 7.47 (dd, *J* = 9.0, 2.2 Hz, 1H), 7.18 – 7.10 (m, 2H), 6.91 – 6.85 (m, 1H), 6.64 (d, *J* = 5.6 Hz, 1H), 3.98 (s, 2H), 2.81 (q, *J* = 7.2 Hz, 2H), 1.23 (t, *J* = 7.2 Hz, 3H). **^{13}C NMR (126 MHz, Chloroform-*d*)** δ 156.62, 151.82, 149.36, 135.24, 128.70, 125.78, 125.64, 125.22, 123.75, 121.05, 117.43, 101.25, 52.26, 43.08, 14.82. **HRMS:** Calculated for $C_{18}H_{19}ClN_3O$ [M+H$^+$]=328.1211, observed =328.1190

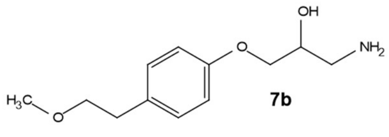

7b

^{1}H NMR (500 MHz, Chloroform-*d*) δ 7.18 – 7.11 (m, 2H), 6.90 – 6.82 (m, 2H), 4.05 – 3.99 (m, 1H), 3.99 – 3.93 (m, 2H), 3.58 (t, *J* = 7.1 Hz, 2H), 3.37 (s, 3H), 3.01 (dd, *J* = 12.9, 3.9 Hz, 1H), 2.90 (dd, *J* = 12.9, 7.2 Hz, 1H), 2.84 (t, *J* = 7.1 Hz, 2H), 2.67 (s, 2H). **^{13}C NMR (126 MHz, Chloroform-*d*)** δ 157.08, 131.54, 129.83, 114.50, 73.82, 70.08, 58.65, 43.92, 35.29. **HRMS:** Calculated for $C_{12}H_{20}NO_3$ [M+H$^+$]=226.1438, observed =226.1424.

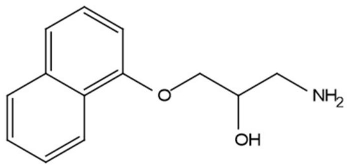

8b

^{1}H NMR (500 MHz, Methanol-*d*$_4$) δ 8.57 (s, 1H), 8.51 – 8.24 (m, 2H), 8.00 – 7.79 (m, 2H), 7.64 – 7.44 (m, 4H), 7.43 – 7.36 (m, 1H), 6.98 – 6.91 (m, 1H), 4.43 – 4.34 (m, 1H), 4.30 – 4.16 (m, 2H), 3.24 – 3.14 (m, 1H). **^{13}C NMR (126 MHz, Methanol-*d*$_4$)** δ 168.40, 153.35, 133.98, 126.51, 125.45, 124.93, 124.25, 120.76, 119.80, 104.11, 68.98, 65.61, 41.60. **HRMS:** Calculated for $C_{13}H_{16}NO_2$ [M+H$^+$]=218.1176, observed =218.1173

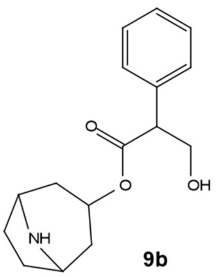

9b

^{1}H NMR (500 MHz, Chloroform-*d*) δ 7.44 – 7.22 (m, 5H), 5.08 (q, *J* = 4.6 Hz, 1H), 4.22 – 4.13 (m, 1H), 3.90 – 3.73 (m, 2H), 3.49 – 3.43 (m, 1H), 3.37 – 3.31 (m, 1H), 2.36 (s, 2H), 2.03 (dd, *J* = 15.0, 4.4 Hz, 1H), 1.95 (dd, *J* = 15.3, 4.2 Hz, 1H), 1.90 – 1.84 (m, 1H), 1.76 (d, *J* = 15.2 Hz, 1H), 1.71 – 1.61 (m, 1H), 1.55 (d, *J* = 15.2 Hz, 1H), 1.52 – 1.43 (m, 1H), 1.34 – 1.26 (m, 1H).. **^{13}C NMR (126 MHz, Chloroform-*d*)** δ 172.36, 135.63, 128.94, 128.18, 127.80, 68.75, 64.20, 54.35, 53.19, 53.10, 37.15, 36.87, 28.89, 28.47. **HRMS:** Calculated for $C_{16}H_{22}NO_3$ [M+H$^+$]=276.1597, observed =276.1594.

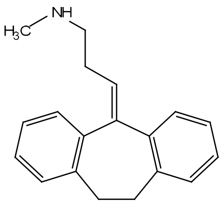

10b

^{1}H NMR (500 MHz, Chloroform-*d*) δ 7.32 – 7.29 (m, 1H), 7.23 – 7.21 (m, 2H), 7.21 – 7.15 (m, 3H), 7.11 – 7.09 (m, 1H), 7.05 (dd, *J* = 6.9, 2.1 Hz, 1H), 5.81 (t, *J* = 7.3 Hz, 1H), 3.33 (t, *J* = 17.5 Hz, 2H), 3.04 – 2.95 (m, 2H), 2.94 – 2.88 (m, 1H), 2.80 (s, 1H), 2.68 – 2.59 (m, 2H), 2.52 (s, 3H). **^{13}C NMR (126 MHz, Chloroform-*d*)** δ 144.46, 141.16, 139.90, 139.27, 137.07, 130.00, 128.70, 128.56, 128.29, 128.08, 127.50, 127.14, 126.05, 125.82, 51.45, 35.84, 33.77, 32.09, 29.46. **HRMS:** Calculated for $C_{19}H_{22}N$ [M+H$^+$]=264.1747, observed =264.1747.

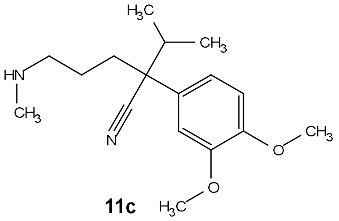

11c

^{1}H NMR (500 MHz, Chloroform-*d*) δ 6.94 (dd, *J* = 8.4, 2.3 Hz, 1H), 6.89 – 6.83 (m, 2H), 3.91 (s, 3H), 3.89 (s, 3H), 2.63 – 2.52 (m, 2H), 2.37 (s, 3H), 2.23 – 2.15 (m, 1H), 2.12 – 2.05 (m, 1H), 1.95 – 1.87 (m, 1H), 1.63 – 1.53 (m, 1H), 1.20 (d, *J* = 6.7 Hz, 3H), 1.19 – 1.14 (m, 1H), 0.81 (d, *J* = 6.7 Hz, 3H). **^{13}C NMR (126 MHz, Chloroform-*d*)** δ 149.04, 148.29, 130.54, 121.37, 118.73, 111.09, 109.53, 56.02, 55.90, 53.36, 51.27, 37.89, 35.91, 35.59, 25.47, 18.97, 18.60. **HRMS:** Calculated for $C_{17}H_{27}N_2O_2$ [M+H$^+$]=291.2067, observed =291.2055

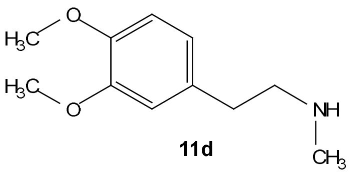

11d

^{1}H NMR (500 MHz, Chloroform-*d*) δ 6.83 (d, *J* = 7.9 Hz, 1H), 6.80 – 6.75 (m, 2H), 3.90 (s, 3H), 3.88 (s, 3H), 2.89 – 2.85 (m, 2H), 2.84 – 2.79 (m, 2H), 2.49 (s, 3H), 2.22 (s, 1H). **^{13}C NMR (126 MHz, Chloroform-*d*)** δ 148.95, 147.52, 132.15, 120.58, 111.95, 111.33, 55.93, 55.85, 53.07, 36.02, 35.37. **HRMS:** Calculated for $C_{11}H_{18}NO_2$ [M+H$^+$]=196.1332, observed =196.1326.

^{1}H and ^{13}C NMR Spectra

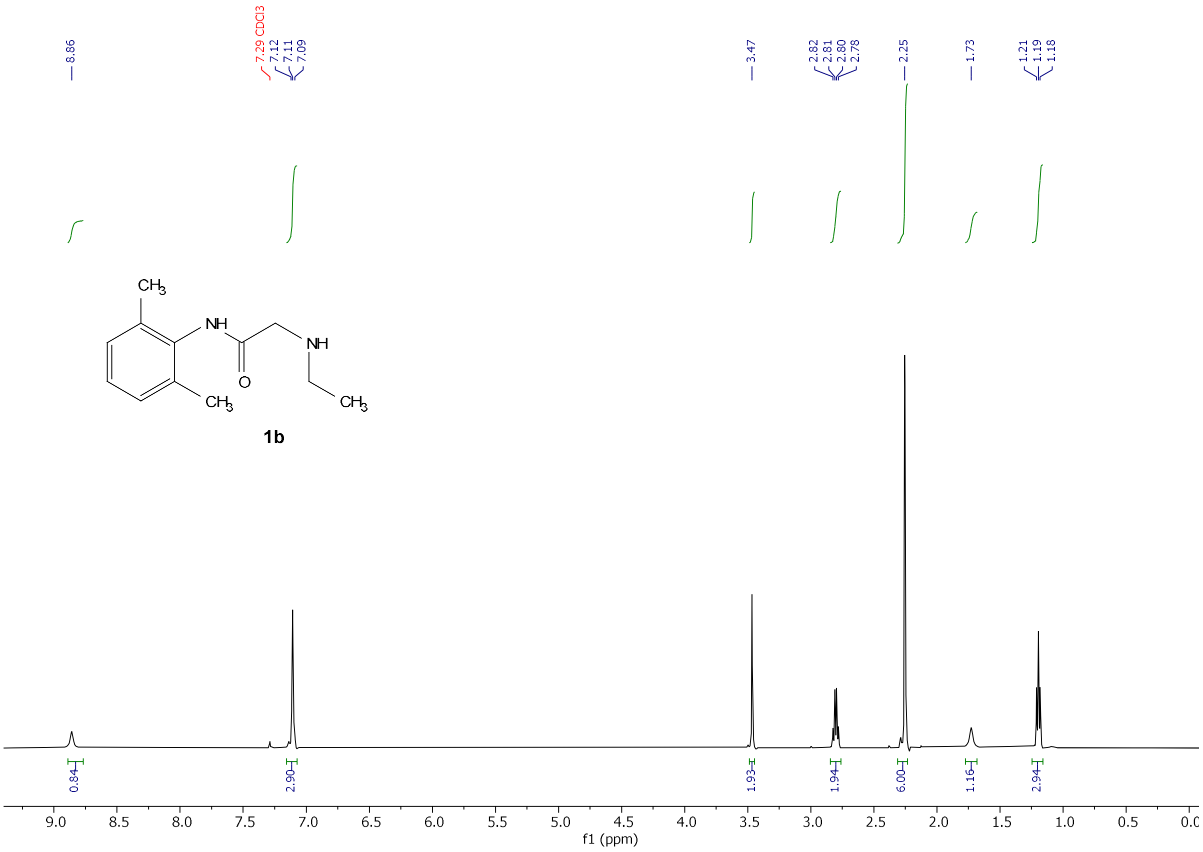

1b

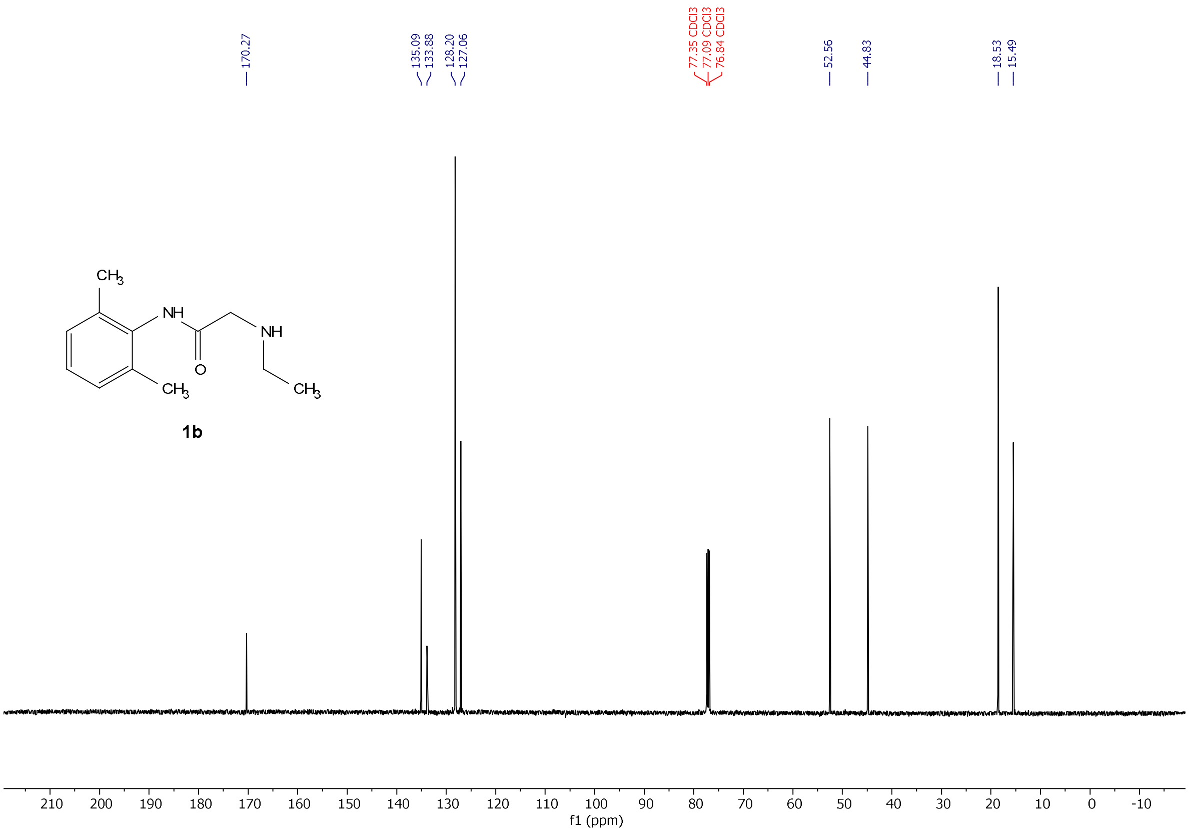

1b

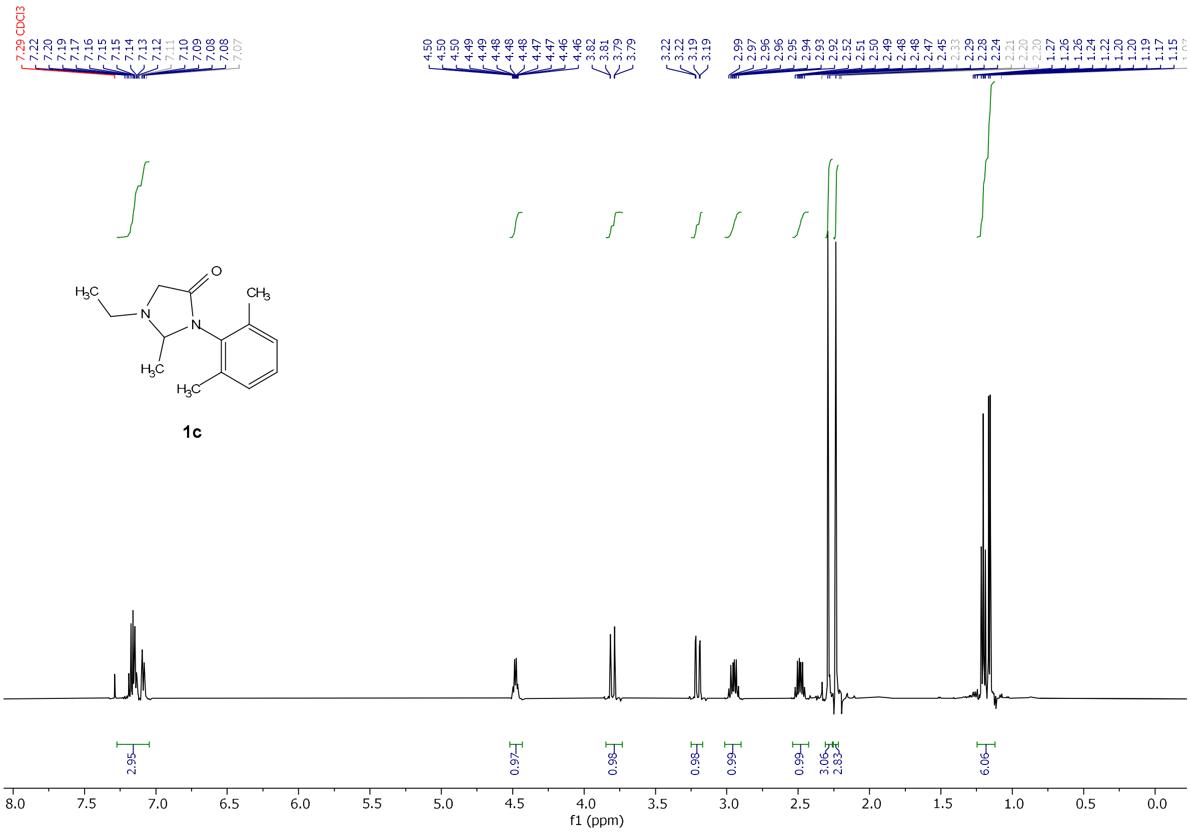

1c

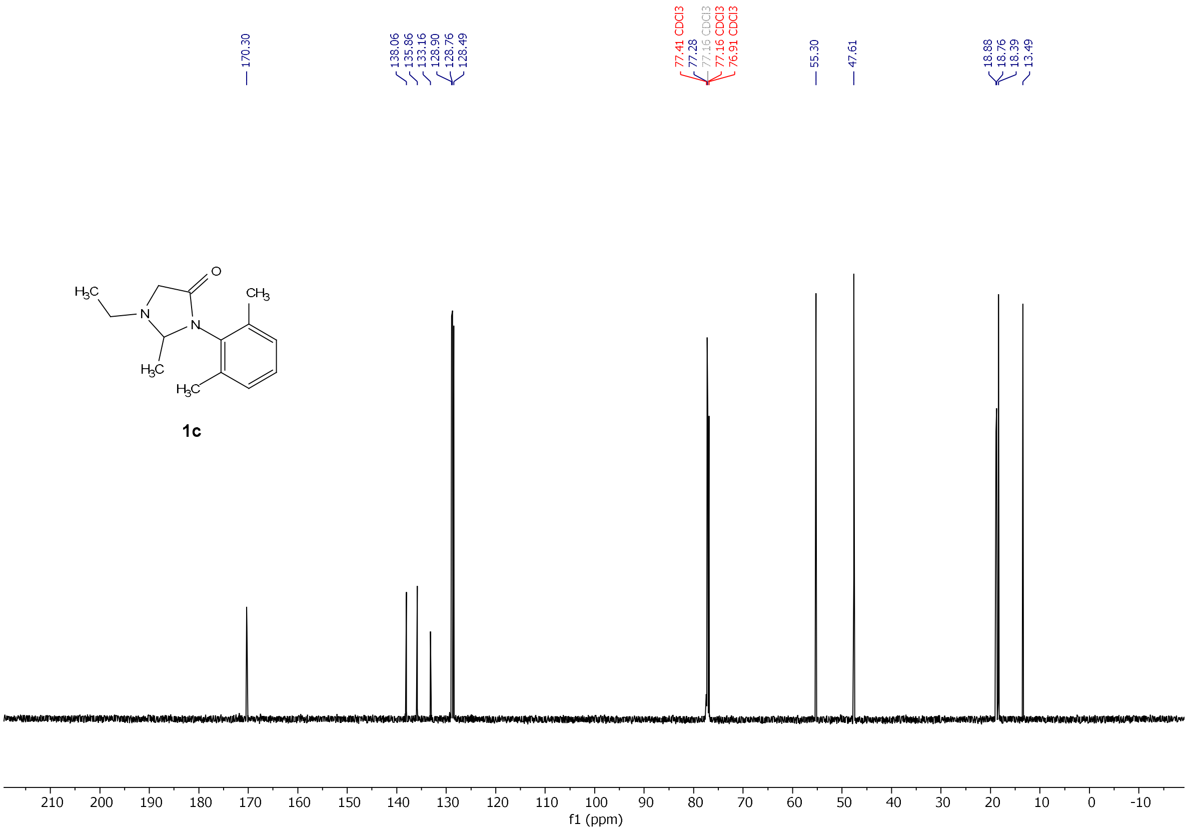

1c

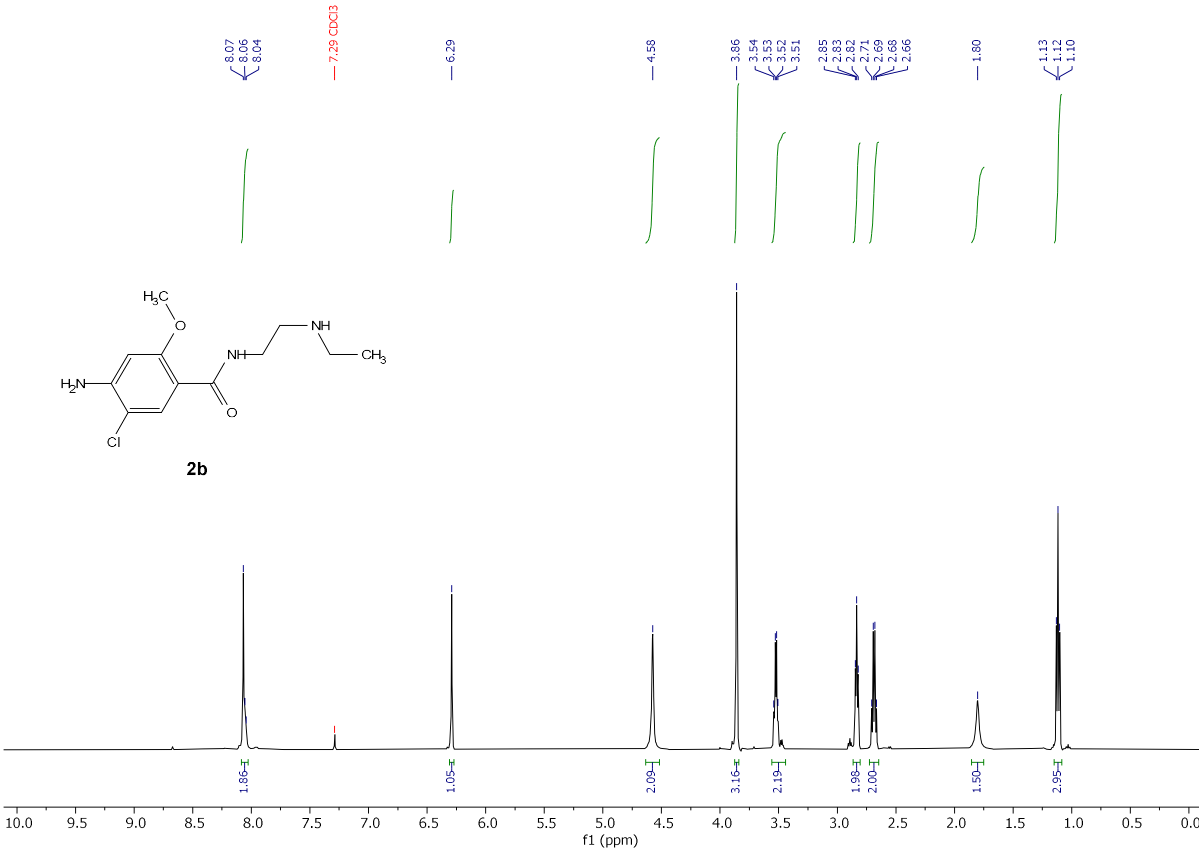
8.07
8.06
8.04
7.29 CDCl3
6.29
4.58
3.86
3.54
3.53
3.52
3.51
2.85
2.83
2.82
2.71
2.69
2.68
2.66
1.80
1.13
1.12
1.10
1.86
1.05
2.09
3.16
2.19
1.98
2.00
1.50
2.95
f1 (ppm)
2b

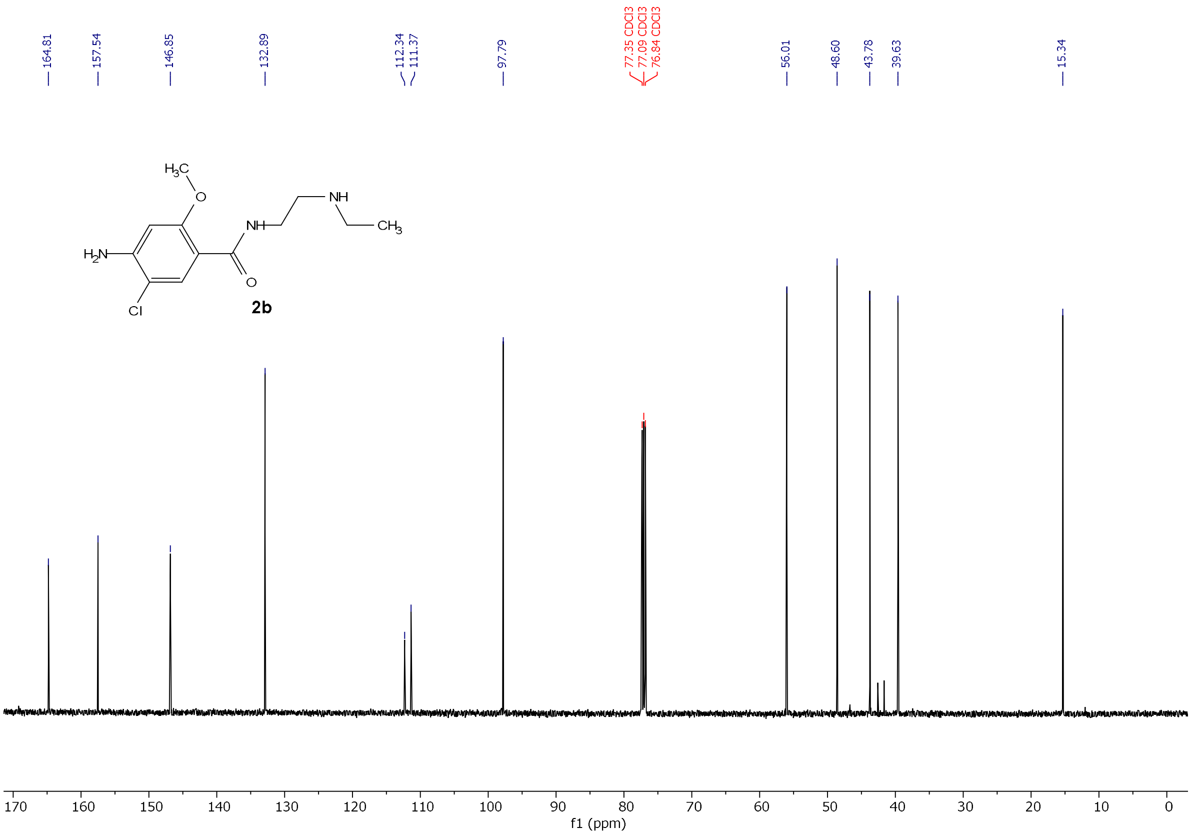
164.81
157.54
146.85
132.89
112.34
111.37
97.79
77.35 CDCl3
77.09 CDCl3
76.84 CDCl3
56.01
48.60
43.78
39.63
15.34
f1 (ppm)
2b

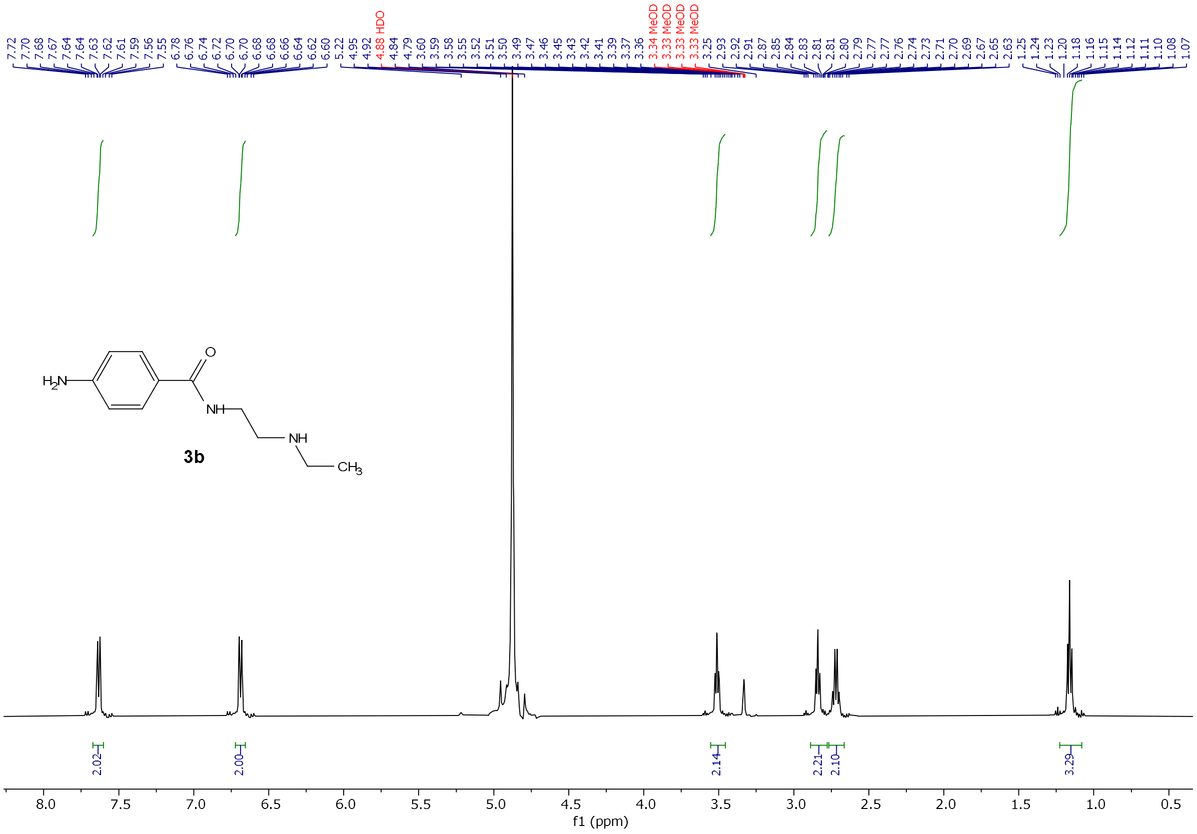

7.72
7.70
7.68
7.67
7.64
7.64
7.63
7.62
7.61
7.59
7.56
7.55
6.78
6.76
6.74
6.72
6.70
6.70
6.68
6.68
6.66
6.64
6.62
6.60
5.22
4.95
4.92
4.88 HDO
4.84
4.79
3.60
3.59
3.58
3.55
3.52
3.51
3.50
3.49
3.47
3.46
3.45
3.43
3.42
3.41
3.39
3.37
3.36
3.34 MeOD
3.33 MeOD
3.33 MeOD
3.33 MeOD
3.25
2.93
2.92
2.91
2.87
2.85
2.84
2.83
2.81
2.81
2.80
2.79
2.77
2.77
2.76
2.74
2.73
2.71
2.70
2.69
2.67
2.65
2.63
1.25
1.24
1.23
1.20
1.18
1.16
1.15
1.14
1.12
1.11
1.10
1.08
1.07
H2N
O
NH
NH
CH3
3b
2.02
2.00
2.14
2.21
2.10
3.29
8.0
7.5
7.0
6.5
6.0
5.5
5.0
4.5
4.0
3.5
3.0
2.5
2.0
1.5
1.0
0.5
f1 (ppm)

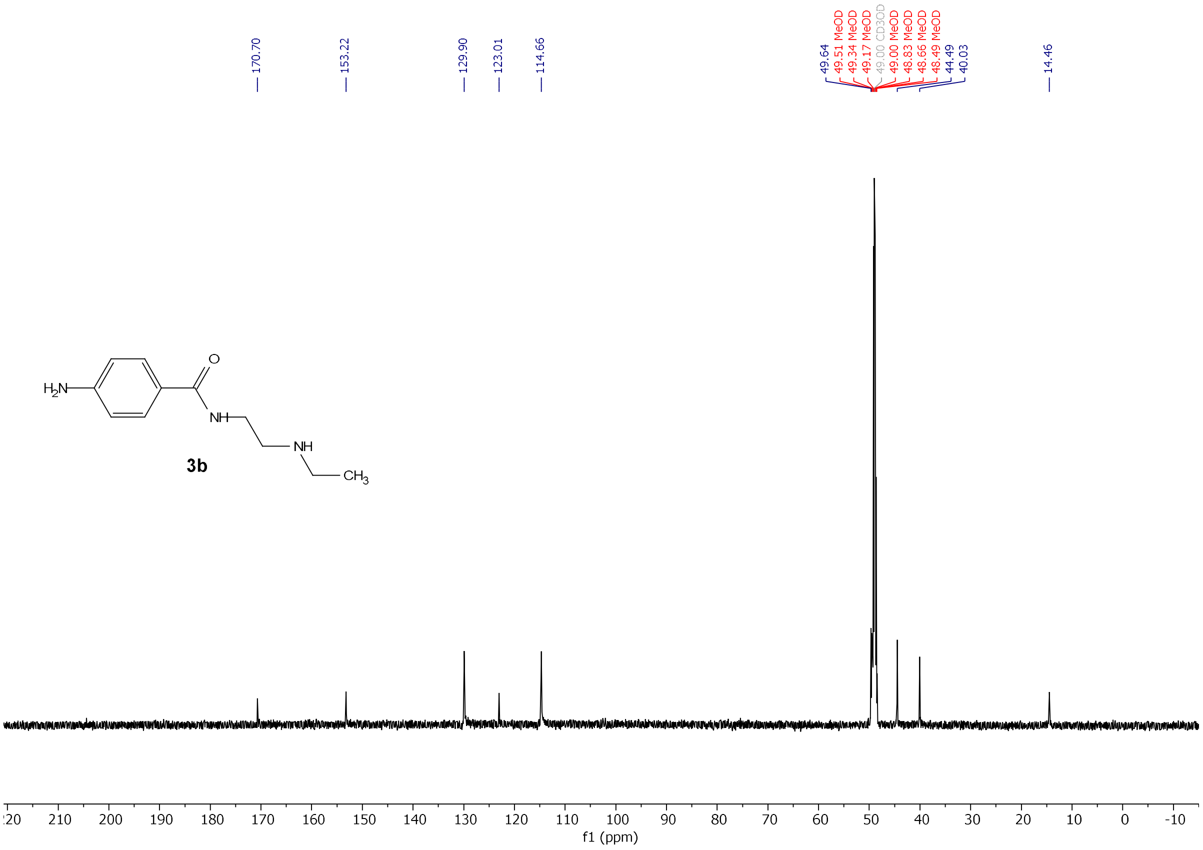

170.70
153.22
129.90
123.01
114.66
49.64
49.51 MeOD
49.34 MeOD
49.17 MeOD
49.00 CD3OD
49.00 MeOD
48.83 MeOD
48.66 MeOD
48.49 MeOD
44.49
40.03
14.46
H2N
O
NH
NH
CH3
3b
210
200
190
180
170
160
150
140
130
120
110
100
90
80
70
60
50
40
30
20
10
0
-10
f1 (ppm)

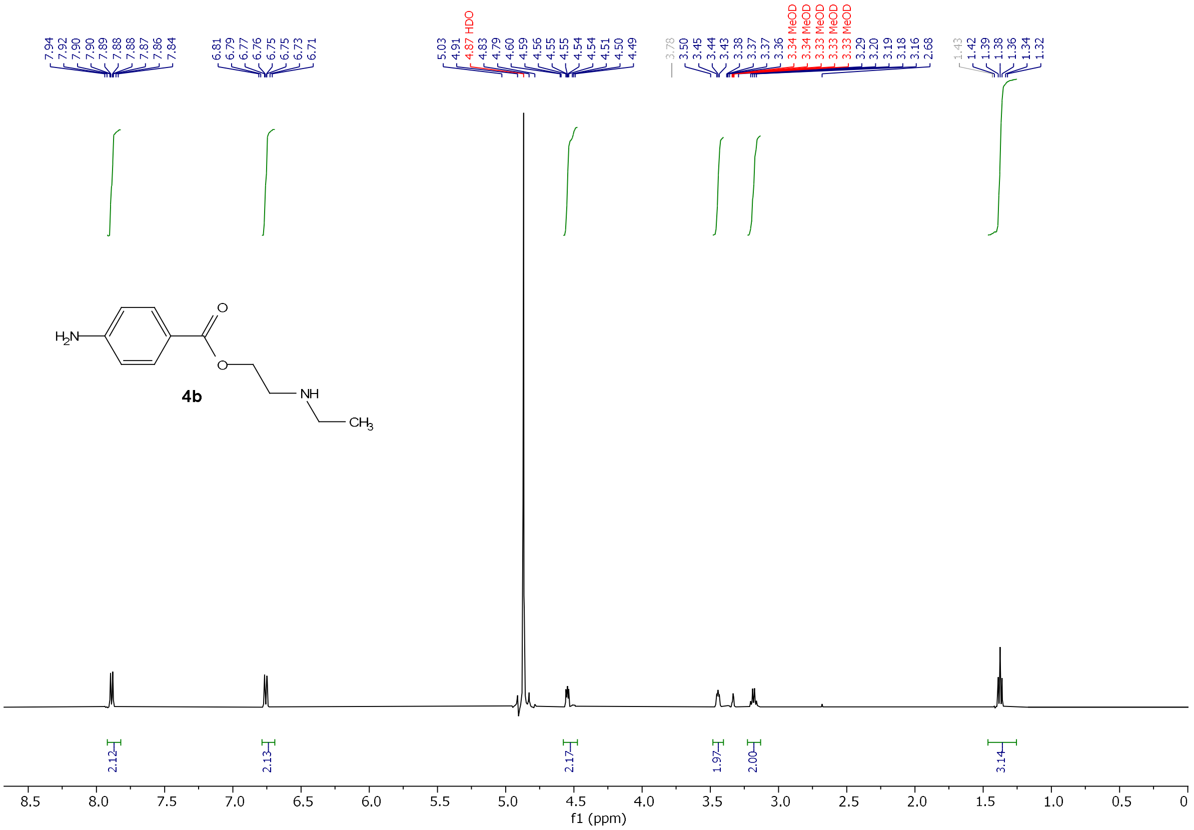
7.94 7.92 7.90 7.90 7.89 7.88 7.88 7.87 7.86 7.84
6.81 6.79 6.77 6.76 6.75 6.75 6.73 6.71
5.03 4.91 4.87 HDO 4.83 4.79 4.60 4.59 4.56 4.55 4.55 4.54 4.54 4.51 4.50 4.49
3.78 3.50 3.45 3.44 3.43 3.38 3.37 3.37 3.36 3.34 MeOD 3.34 MeOD 3.33 MeOD 3.33 MeOD 3.33 MeOD 3.29 3.20 3.19 3.18 3.16 2.68
1.43 1.42 1.39 1.38 1.36 1.34 1.32
H_2N
O
O
NH
CH_3
4b
2.12 2.13 2.17 1.97 2.00 3.14
8.5 8.0 7.5 7.0 6.5 6.0 5.5 5.0 4.5 4.0 3.5 3.0 2.5 2.0 1.5 1.0 0.5 0
f1 (ppm)

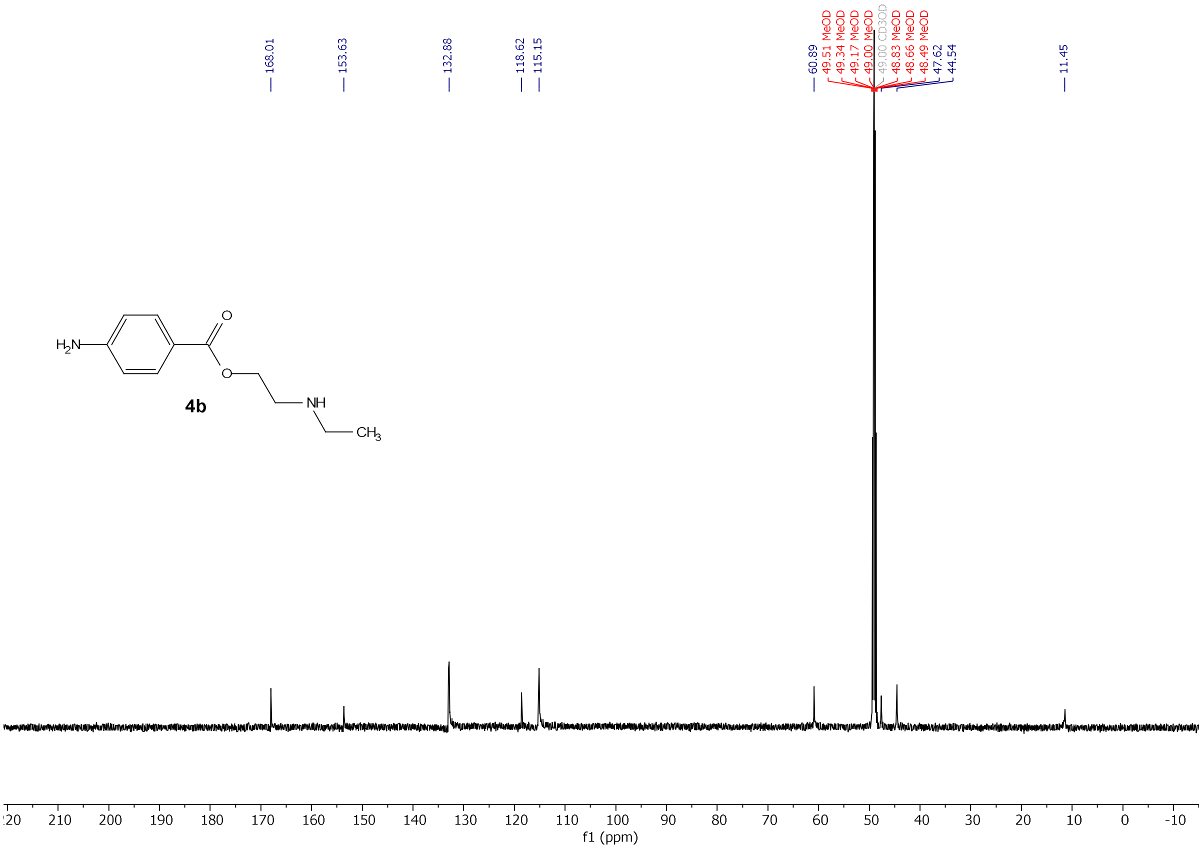
168.01
153.63
132.88
118.62
115.15
60.89 49.51 MeOD 49.34 MeOD 49.17 MeOD 49.00 MeOD 49.00 CD3OD 48.83 MeOD 48.66 MeOD 48.49 MeOD 47.62 44.54
11.45
H_2N
O
O
NH
CH_3
4b
220 210 200 190 180 170 160 150 140 130 120 110 100 90 80 70 60 50 40 30 20 10 0 -10
f1 (ppm)

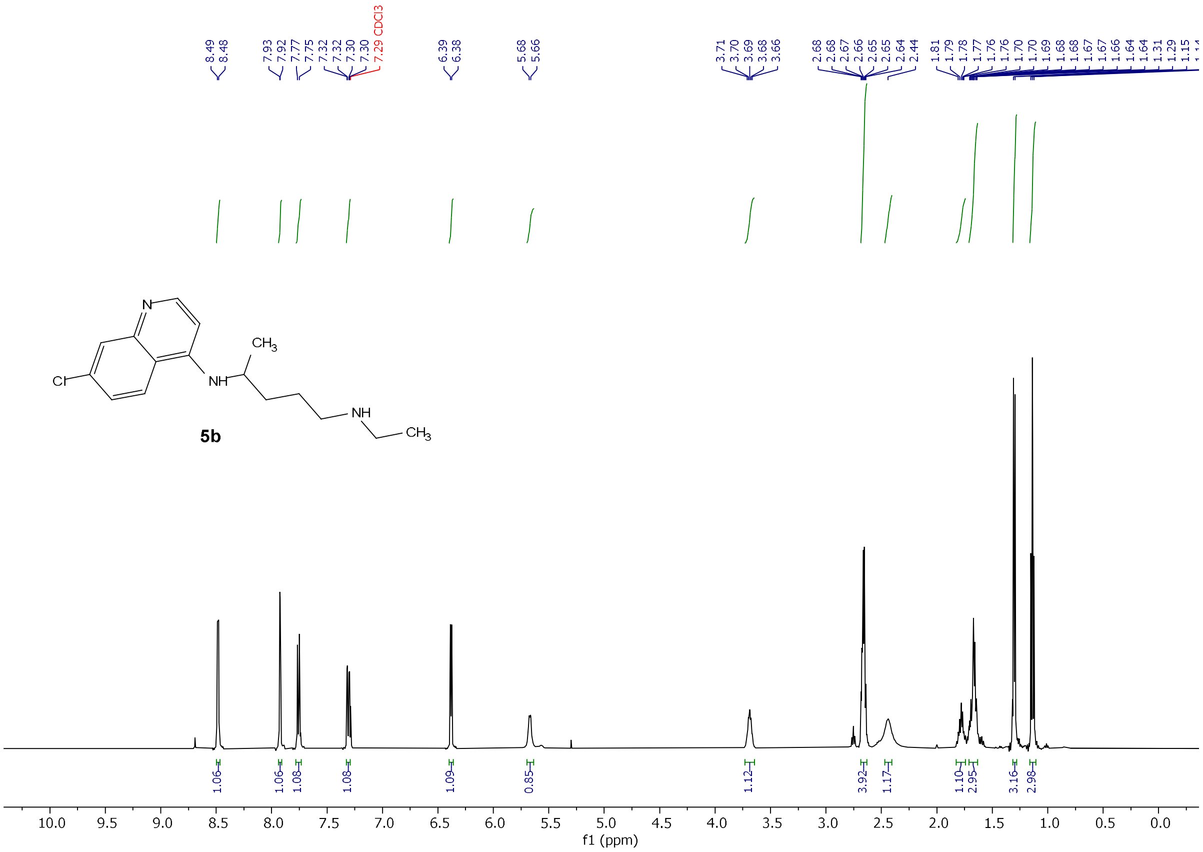

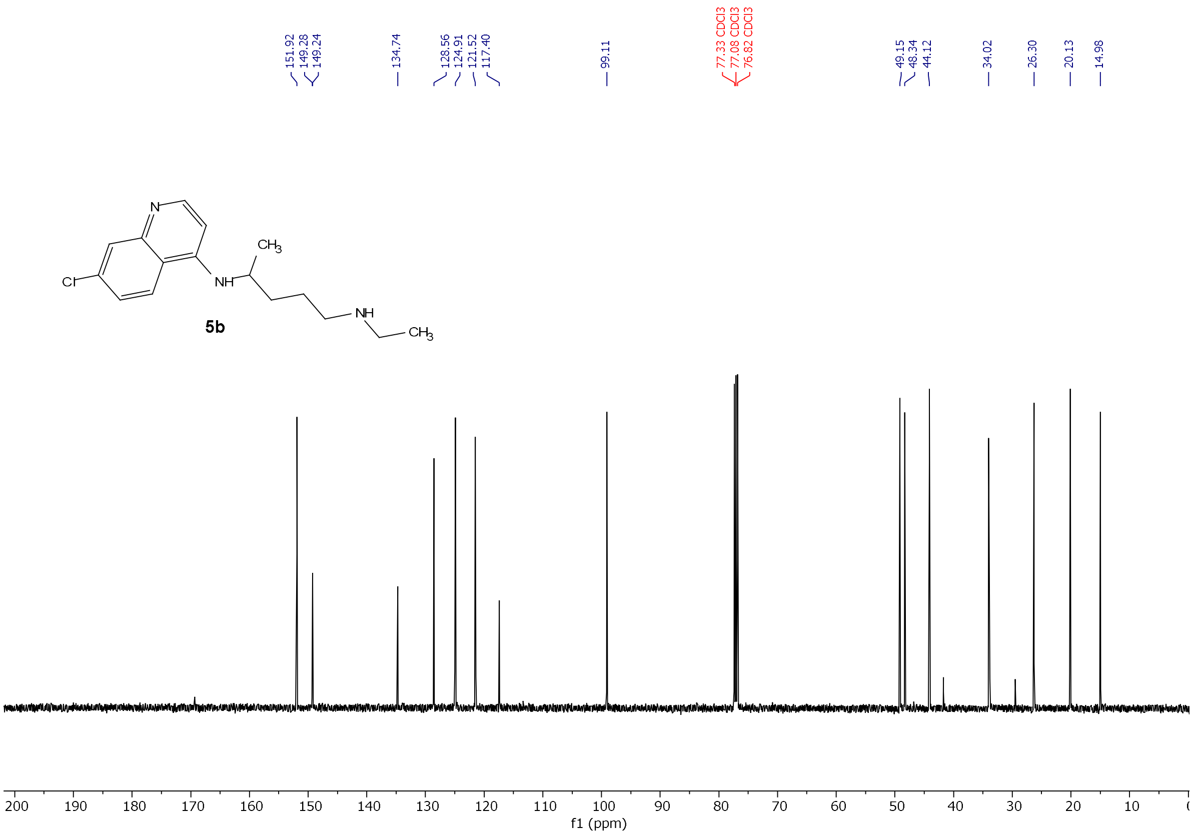

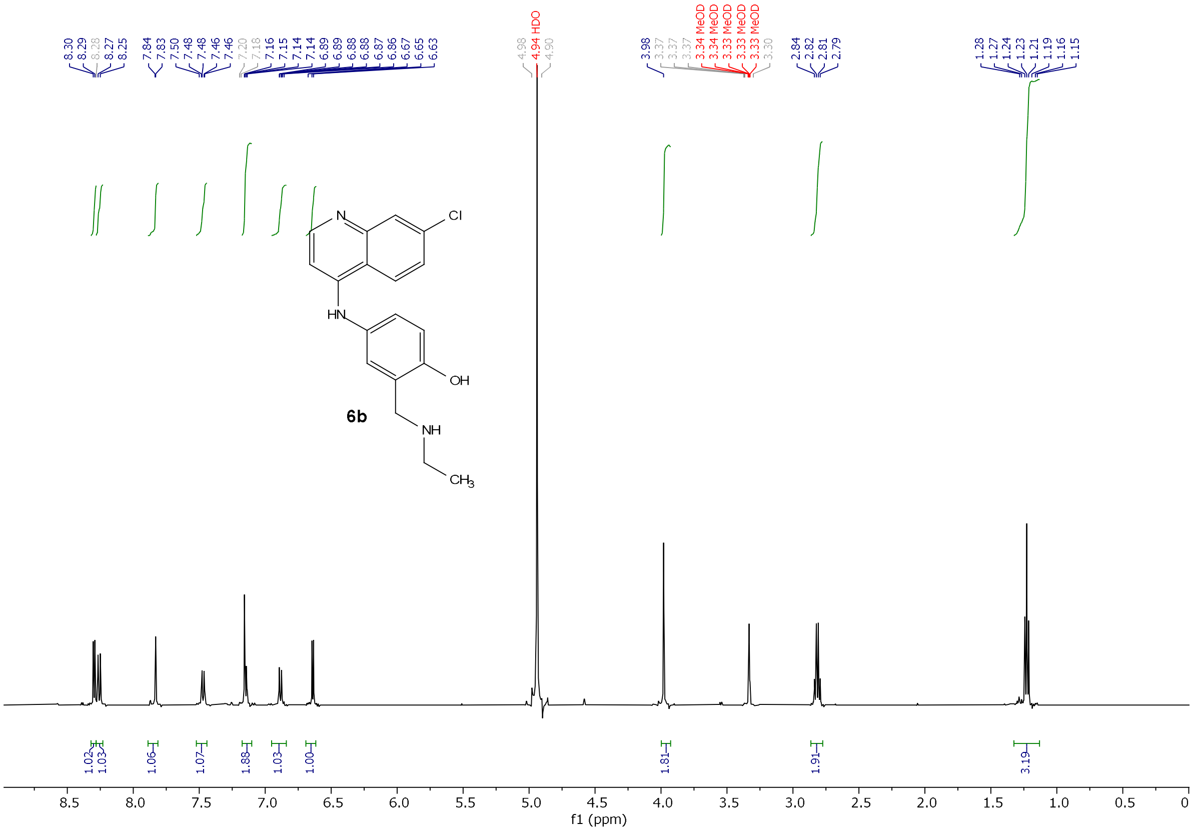
8.30
8.29
8.28
8.27
8.25
7.84
7.83
7.50
7.48
7.48
7.46
7.46
7.20
7.18
7.16
7.15
7.14
7.14
6.89
6.89
6.88
6.88
6.87
6.86
6.67
6.65
6.63
4.98
4.94 HDO
4.90
3.98
3.37
3.37
3.37
3.34 MeOD
3.34 MeOD
3.33 MeOD
3.33 MeOD
3.33 MeOD
3.30
2.84
2.82
2.81
2.79
1.28
1.27
1.24
1.23
1.21
1.19
1.16
1.15
N
Cl
HN
OH
6b
NH
CH3
1.02
1.03
1.06
1.07
1.88
1.03
1.00
1.81
1.91
3.19
8.5 8.0 7.5 7.0 6.5 6.0 5.5 5.0 4.5 4.0 3.5 3.0 2.5 2.0 1.5 1.0 0.5 0
f1 (ppm)

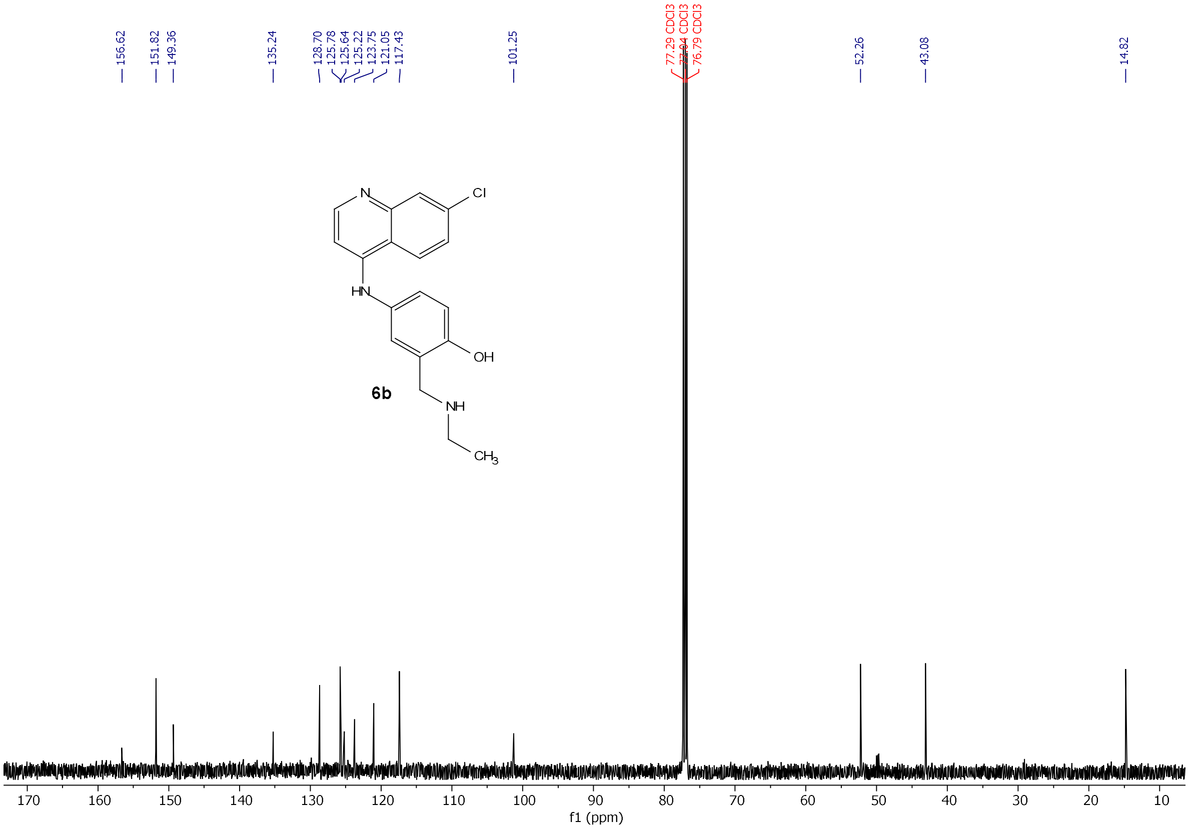
156.62
151.82
149.36
135.24
128.70
125.78
125.64
125.22
123.75
121.05
117.43
101.25
77.29 CDCl3
77.04 CDCl3
76.79 CDCl3
52.26
43.08
14.82
N
Cl
HN
OH
6b
NH
CH3
170 160 150 140 130 120 110 100 90 80 70 60 50 40 30 20 10
f1 (ppm)

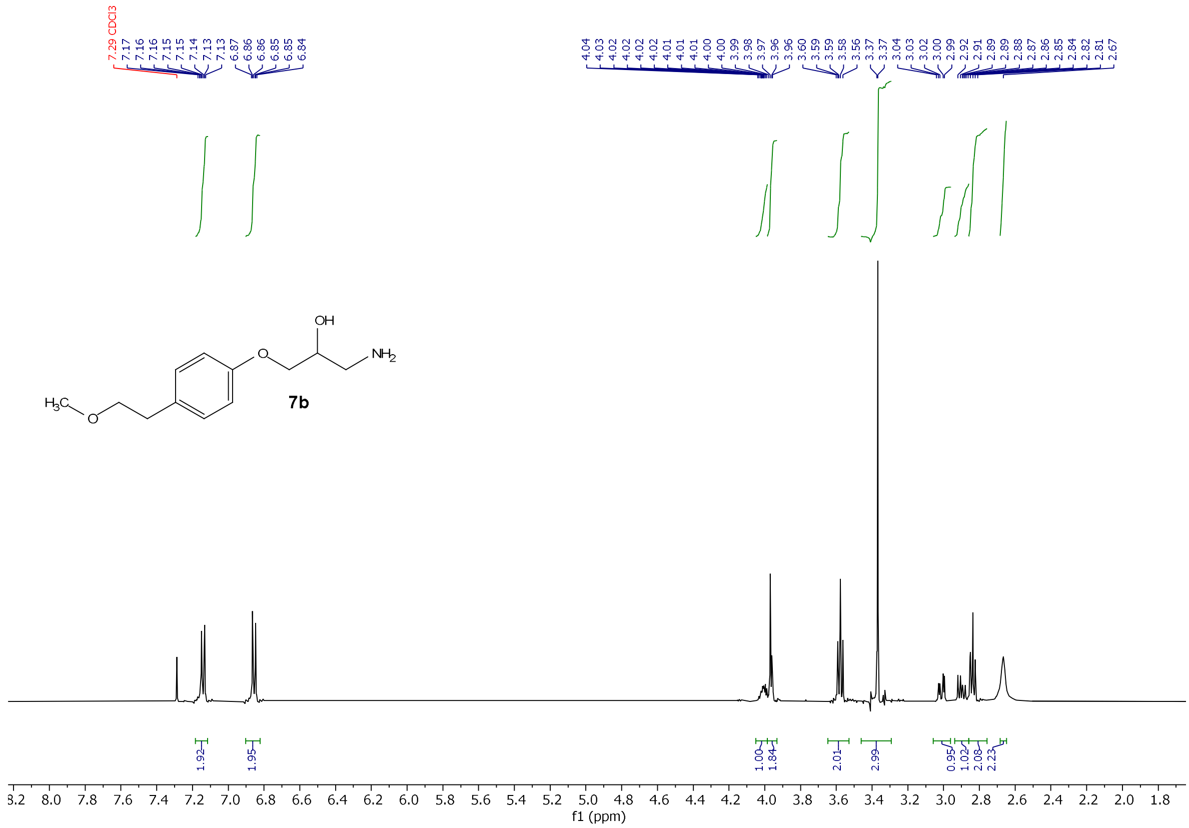
7.29 CDCl3
7.17
7.16
7.16
7.15
7.14
7.13
7.13
6.87
6.86
6.86
6.85
6.85
6.84
4.04
4.03
4.02
4.02
4.02
4.02
4.01
4.01
4.01
4.00
4.00
3.99
3.98
3.97
3.96
3.96
3.60
3.59
3.59
3.58
3.56
3.37
3.37
3.04
3.03
3.02
3.00
2.99
2.92
2.91
2.89
2.89
2.88
2.87
2.86
2.85
2.84
2.82
2.81
2.67
OH
NH2
H3C
O
O
7b
1.92
1.95
1.00
1.84
2.01
2.99
0.95
1.02
2.08
2.23
f1 (ppm)

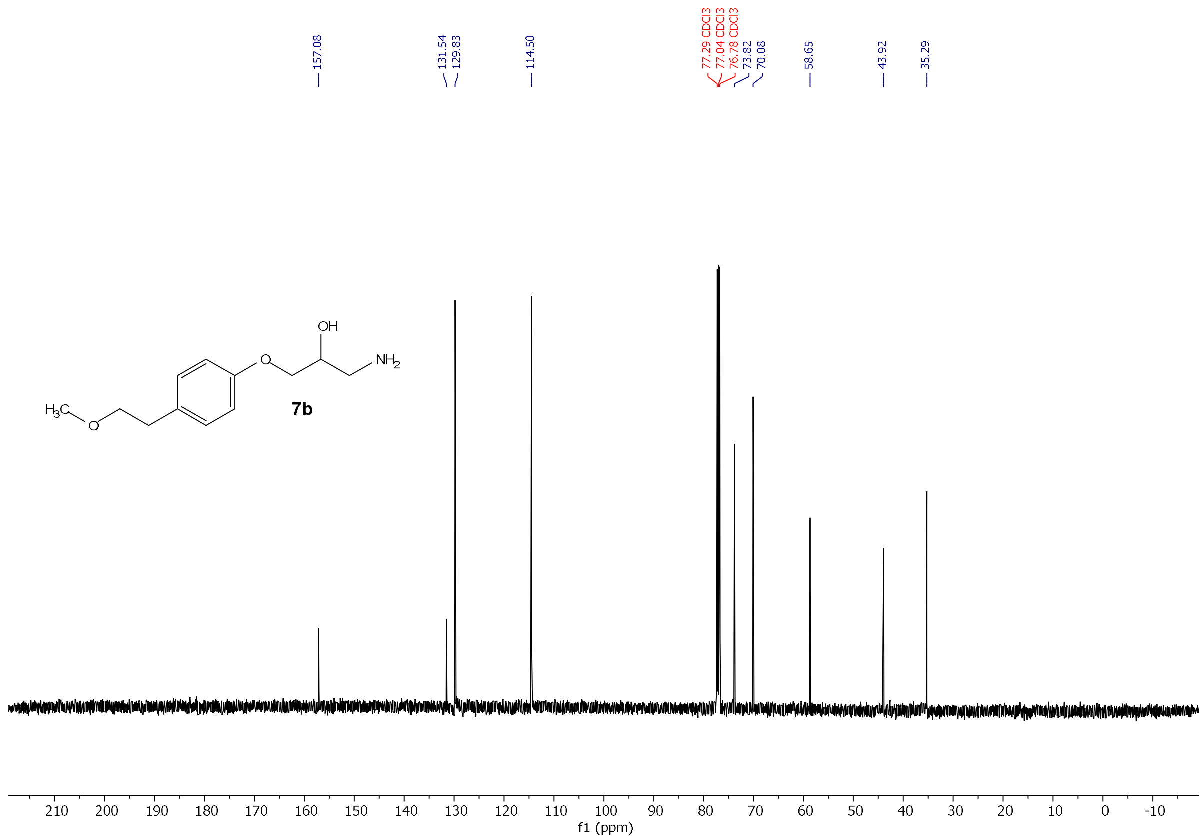
157.08
131.54
129.83
114.50
77.29 CDCl3
77.04 CDCl3
76.78 CDCl3
73.82
70.08
58.65
43.92
35.29
OH
NH2
H3C
O
O
7b
f1 (ppm)

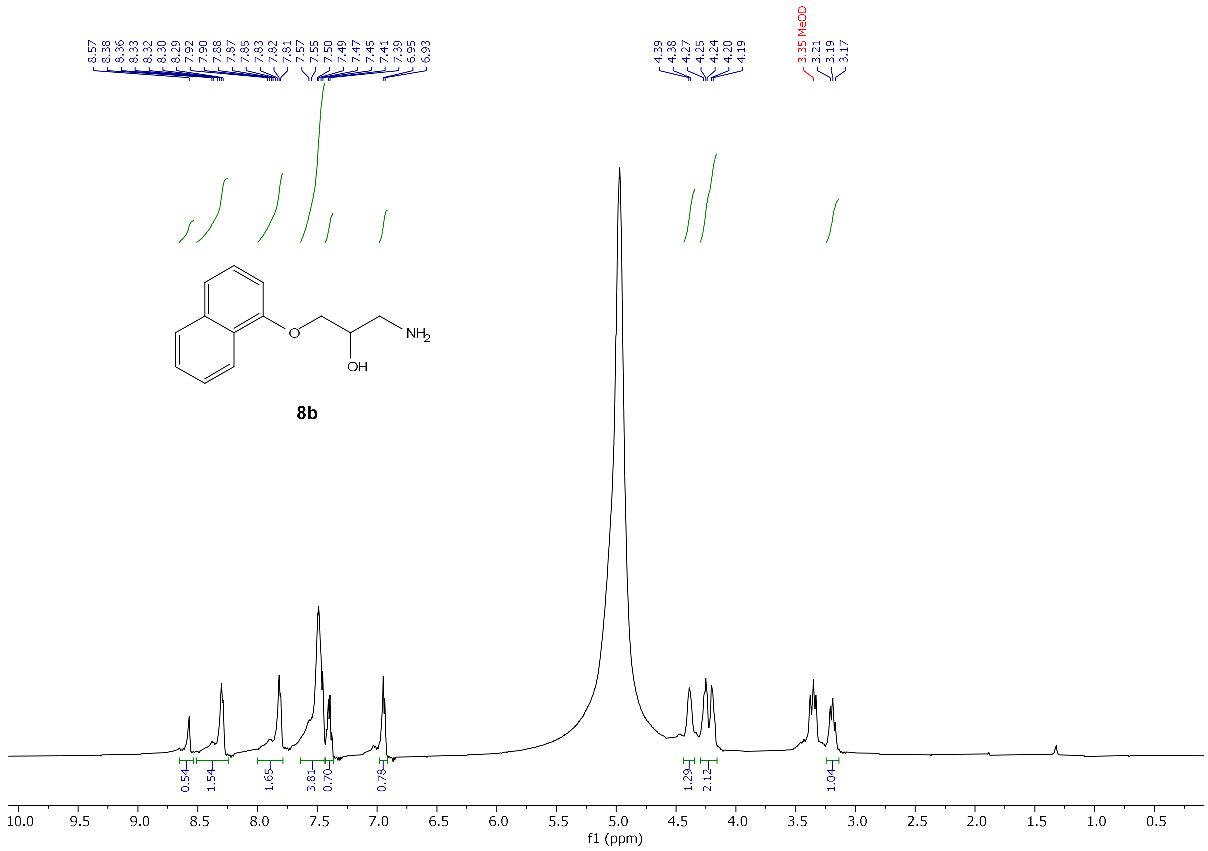
8.57
8.38
8.36
8.33
8.32
8.30
8.29
7.92
7.90
7.88
7.87
7.85
7.83
7.82
7.81
7.57
7.55
7.50
7.49
7.47
7.45
7.41
7.39
6.95
6.93
4.39
4.38
4.27
4.25
4.24
4.20
4.19
3.35 MeOD
3.21
3.19
3.17
O
NH2
OH
8b
0.54
1.54
1.65
3.81
0.70
0.78
1.29
2.12
1.04
f1 (ppm)

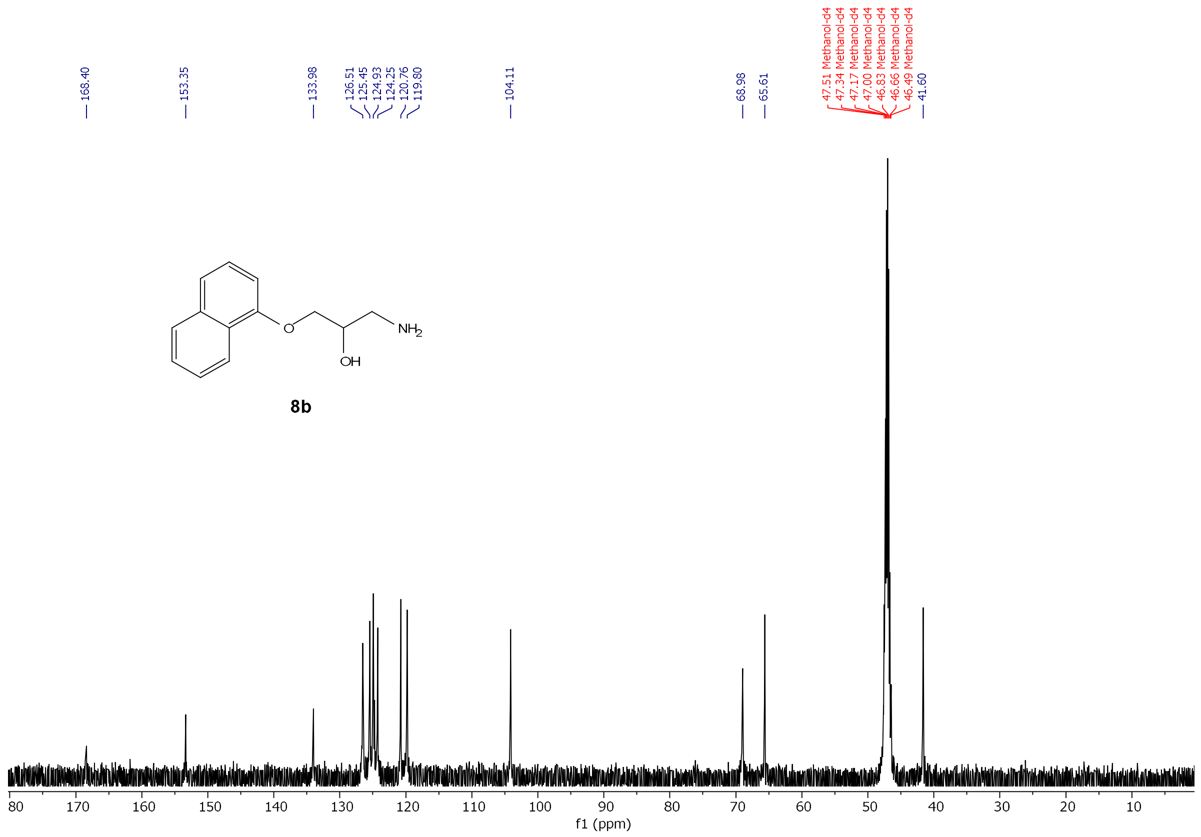
168.40
153.35
133.98
126.51
125.45
124.93
124.25
120.76
119.80
104.11
68.98
65.61
47.51 Methanol-d4
47.34 Methanol-d4
47.17 Methanol-d4
47.00 Methanol-d4
46.83 Methanol-d4
46.66 Methanol-d4
46.49 Methanol-d4
41.60
O
NH2
OH
8b
f1 (ppm)

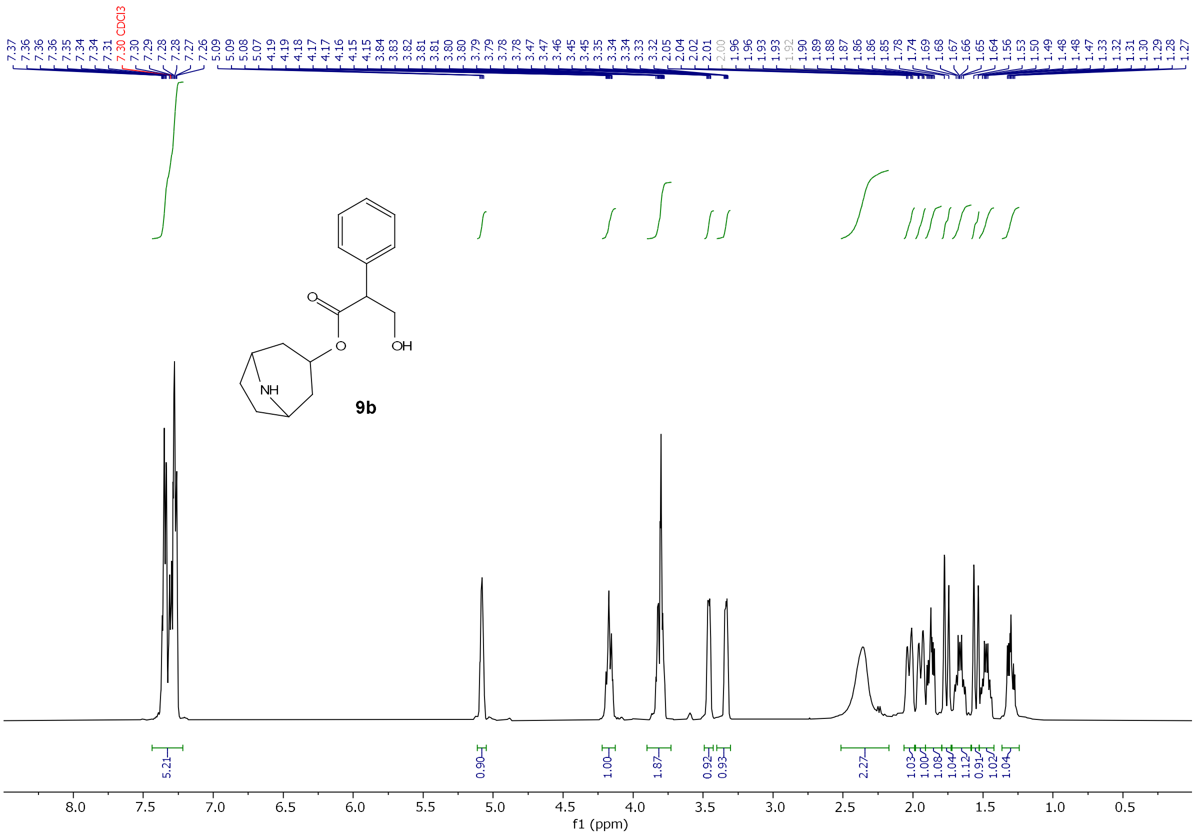
9b
f1 (ppm)

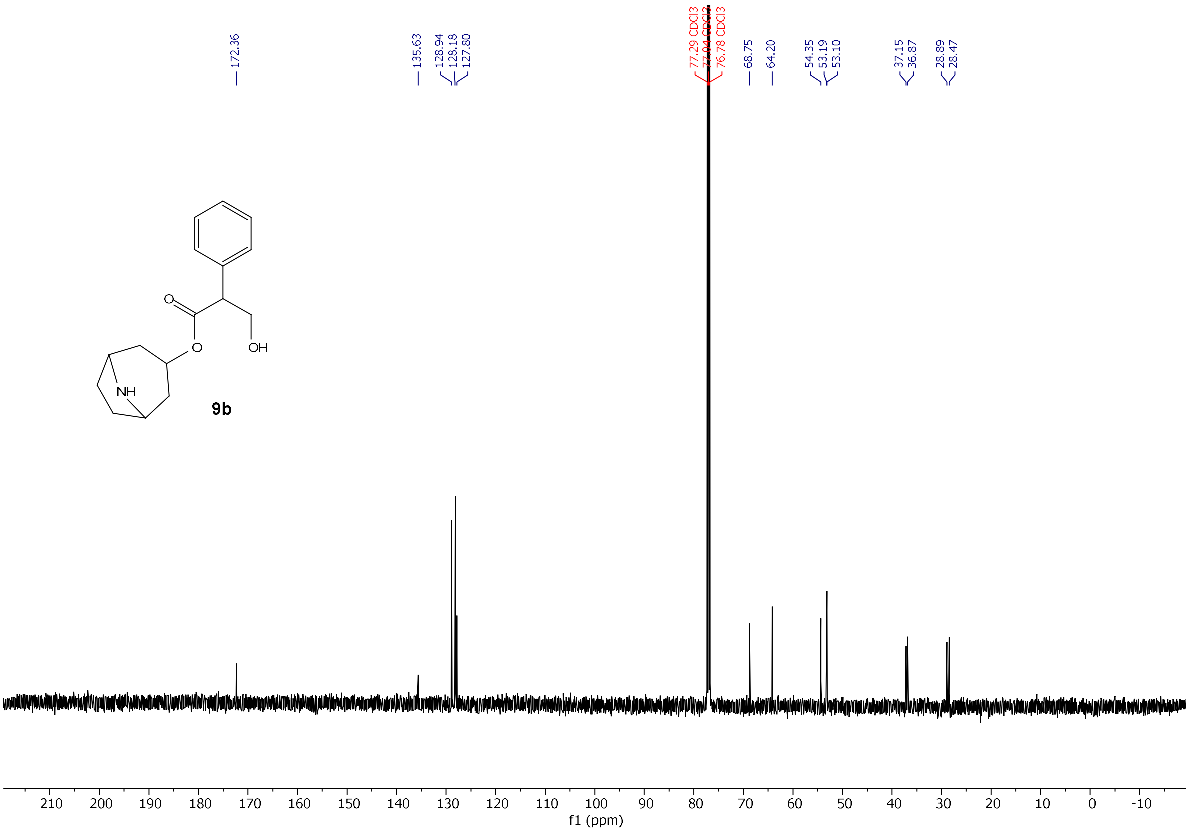
9b
f1 (ppm)

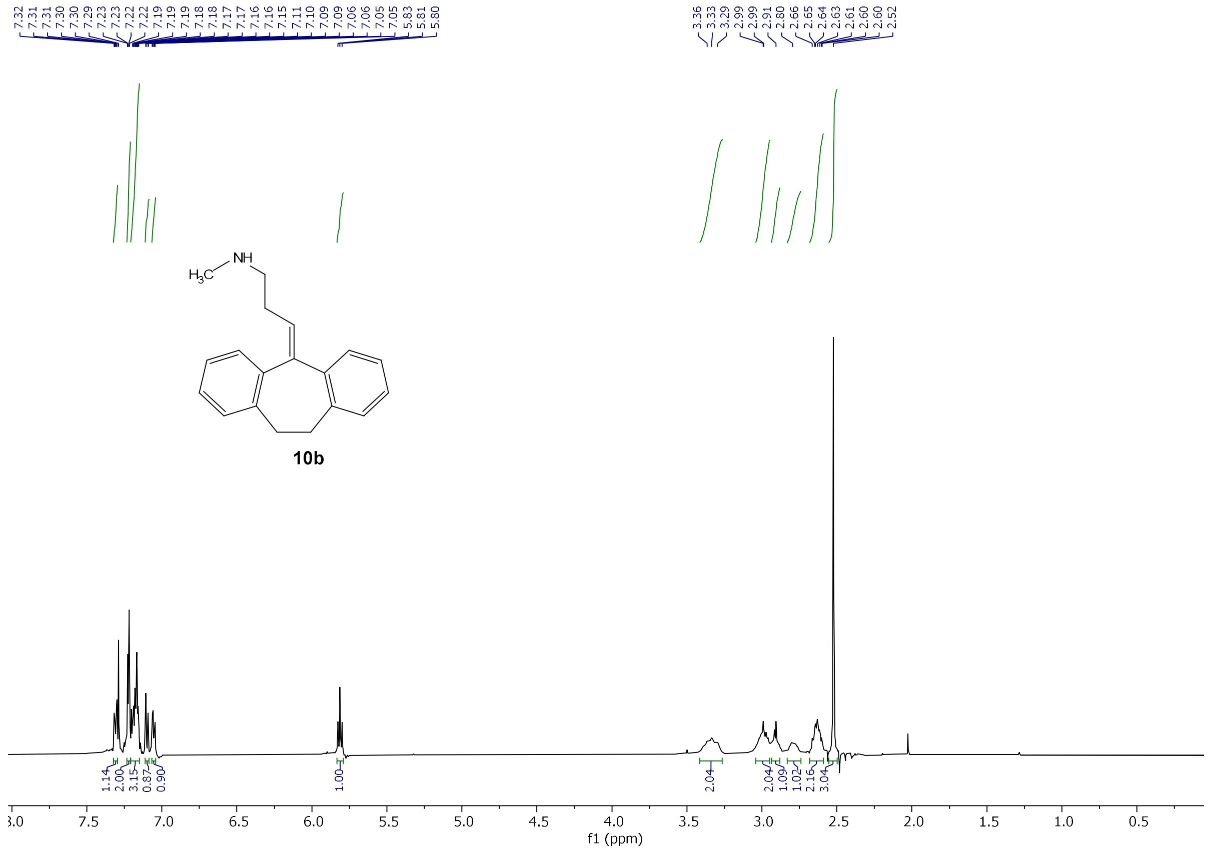
7.32 7.31 7.31 7.30 7.30 7.29 7.23 7.23 7.22 7.22 7.19 7.19 7.19 7.18 7.18 7.17 7.17 7.16 7.16 7.15 7.11 7.10 7.09 7.09 7.06 7.06 7.05 7.05 5.83 5.81 5.80
3.36 3.33 3.29 2.99 2.99 2.91 2.80 2.66 2.65 2.64 2.63 2.61 2.60 2.60 2.52
H3C NH
10b
1.14 2.00 3.15 0.87 0.90
1.00
2.04
2.04 1.09 1.02 2.16 3.04
f1 (ppm)

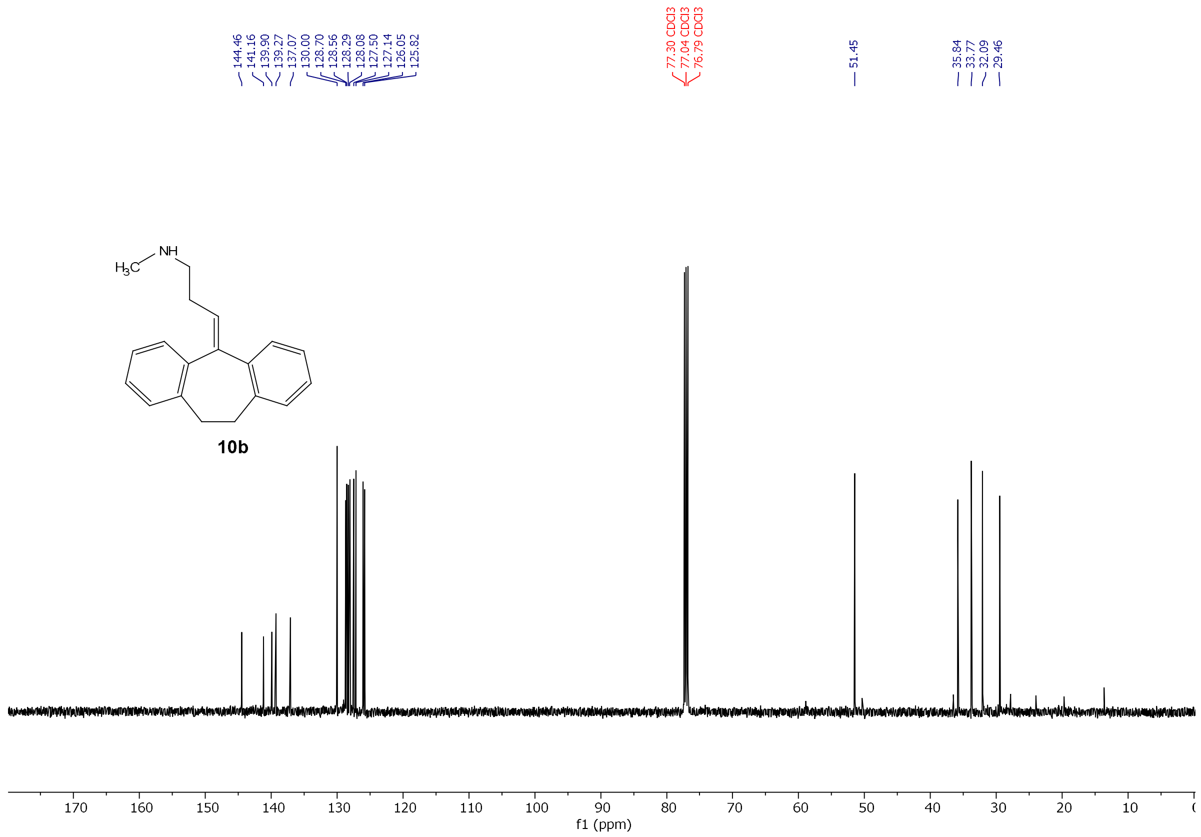
144.46 141.16 139.90 139.27 137.07 130.00 128.70 128.56 128.29 128.08 127.50 127.14 126.05 125.82
77.30 CDCl3 77.04 CDCl3 76.79 CDCl3
51.45
35.84 33.77 32.09 29.46
H3C NH
10b
f1 (ppm)

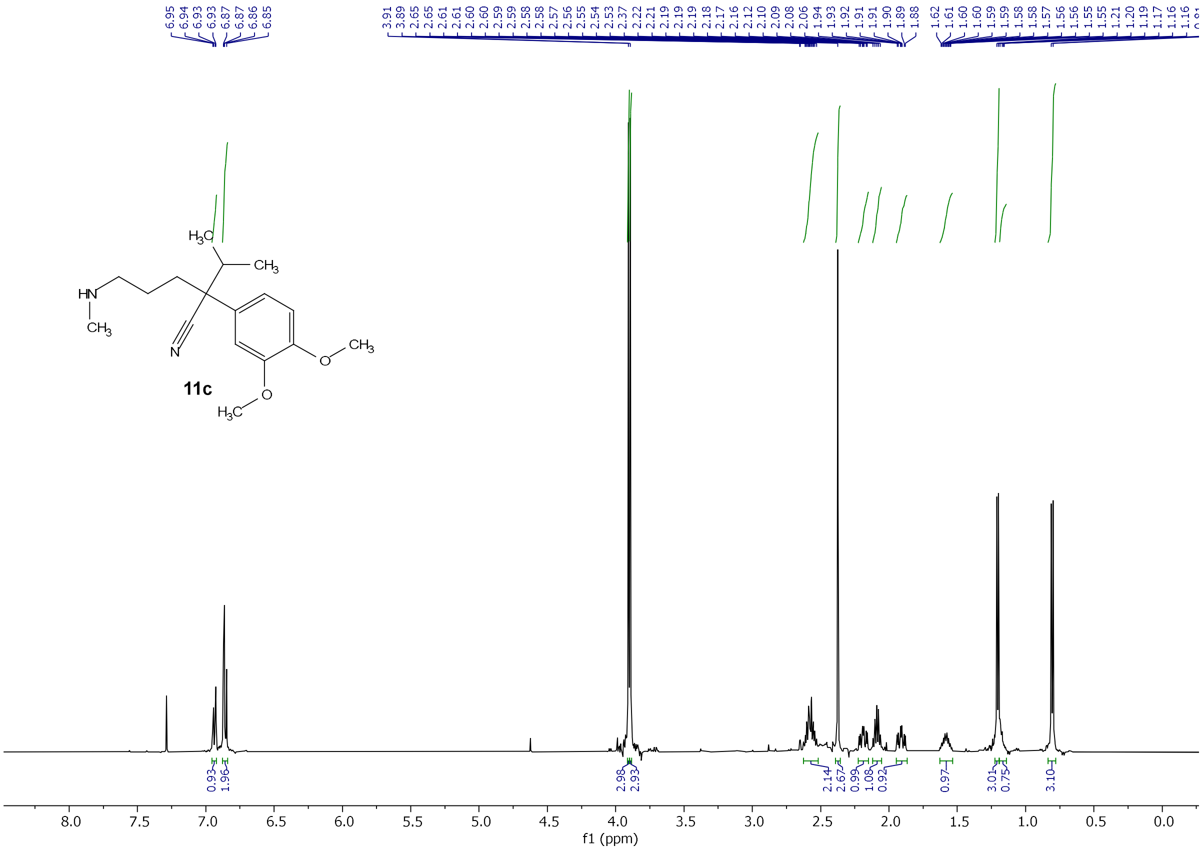
6.95 6.94 6.93 6.93 6.87 6.87 6.86 6.85
3.91 3.89 2.65 2.65 2.61 2.61 2.60 2.60 2.59 2.59 2.58 2.58 2.57 2.56 2.55 2.54 2.53 2.37 2.22 2.21 2.19 2.19 2.19 2.18 2.17 2.16 2.12 2.10 2.09 2.08 2.06 1.94 1.93 1.92 1.91 1.91 1.90 1.89 1.88
1.62 1.61 1.60 1.60 1.59 1.59 1.58 1.58 1.57 1.56 1.56 1.55 1.55 1.21 1.20 1.19 1.17 1.16 1.16
H3C
CH3
HN
CH3
N
O
CH3
O
H3C
11c
0.93 1.96 2.98 2.93 2.14 2.67 0.99 1.08 0.92 0.97 3.01 0.75 3.10
8.0 7.5 7.0 6.5 6.0 5.5 5.0 4.5 4.0 3.5 3.0 2.5 2.0 1.5 1.0 0.5 0.0
f1 (ppm)

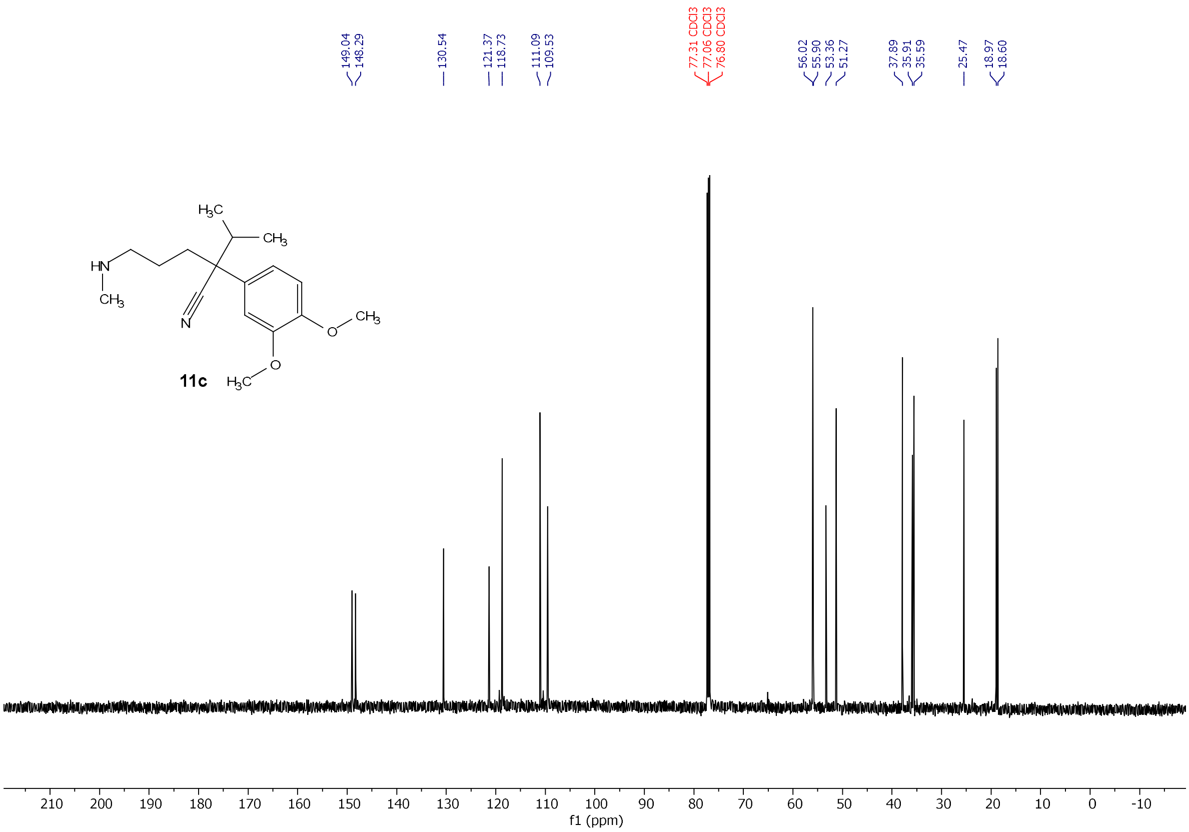
149.04 148.29
130.54
121.37 118.73
111.09 109.53
77.31 CDCl3 77.06 CDCl3 76.80 CDCl3
56.02 55.90 53.36 51.27
37.89 35.91 35.59
25.47
18.97 18.60
H3C
CH3
HN
CH3
N
O
CH3
11c
H3C
O
210 200 190 180 170 160 150 140 130 120 110 100 90 80 70 60 50 40 30 20 10 0 -10
f1 (ppm)

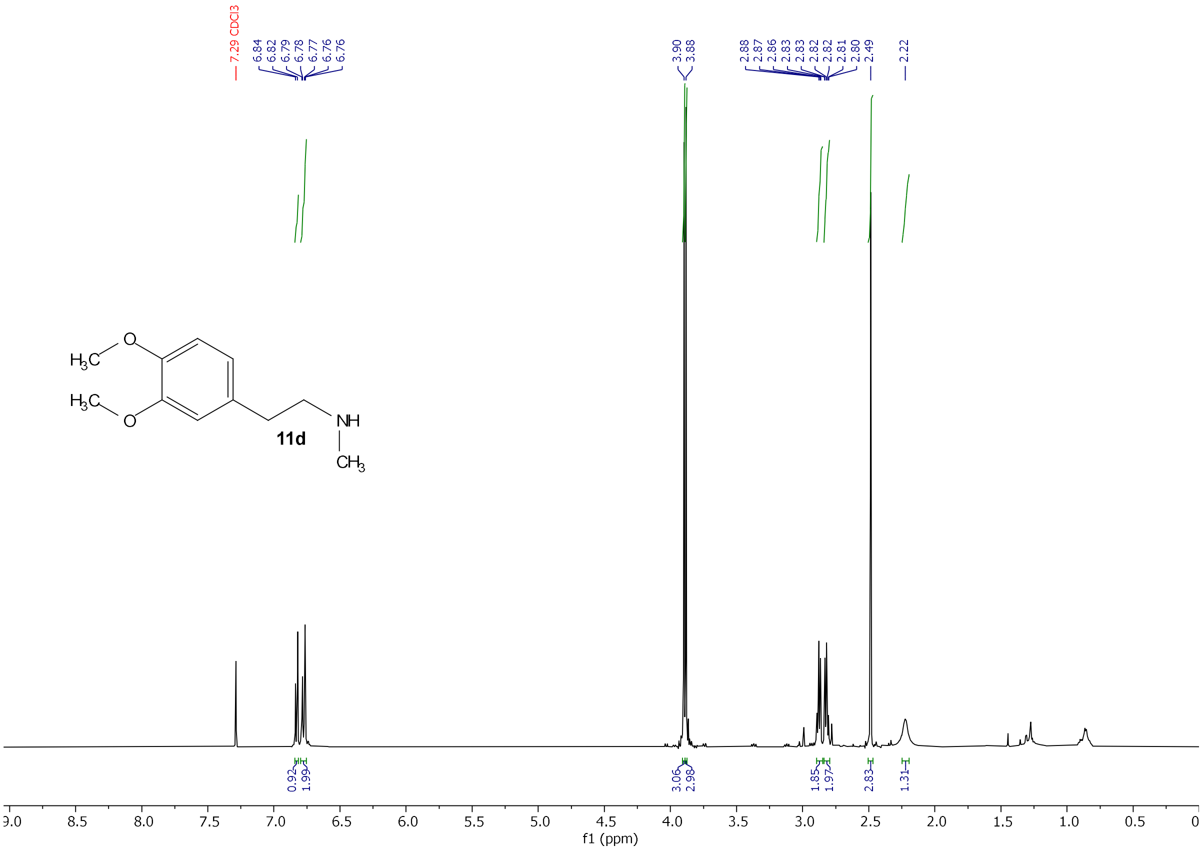
7.29 CDCl3
6.84
6.82
6.79
6.78
6.77
6.76
6.76
3.90
3.88
2.88
2.87
2.86
2.83
2.83
2.82
2.82
2.81
2.80
2.49
2.22
H3C
O
H3C
O
NH
CH3
11d
0.92
1.99
3.06
2.98
1.85
1.97
2.83
1.31
f1 (ppm)

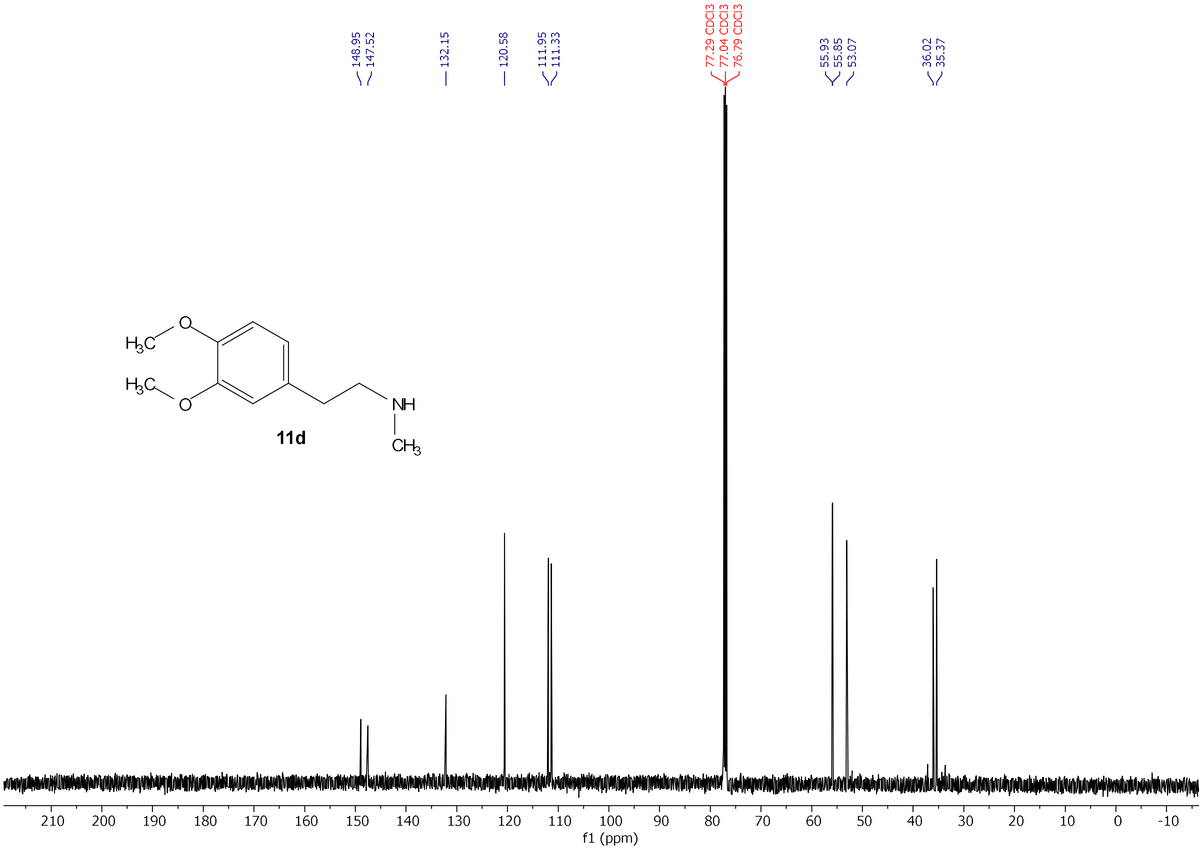
148.95
147.52
132.15
120.58
111.95
111.33
77.29 CDCl3
77.04 CDCl3
76.79 CDCl3
55.93
55.85
53.07
36.02
35.37
H3C
O
H3C
O
NH
CH3
11d
f1 (ppm)

References

[1] D. Steinebrunner, G. Schnurpfeil, D. Wöhrle, A. Wittstock, *RSC Adv.* **2019**, *10*, 53–59.

[2] E. Detsi, E. De Jong, A. Zinchenko, Z. Vuković, I. Vuković, S. Punzhin, K. Loos, G. Ten Brinke, H. A. De Raedt, P. R. Onck, et al., *Acta Mater.* **2011**, *59*, 7488–7497.

[3] Y. H. Tan, J. A. Davis, K. Fujikawa, N. V. Ganesh, A. V. Demchenko, K. J. Stine, *J. Mater. Chem.* **2012**, *22*, 6733–6745.
